# Stromal cell-derived factor 1 regulates *in vitro* sperm migration towards the cumulus-oocyte complex in cattle

**DOI:** 10.1371/journal.pone.0232536

**Published:** 2020-04-30

**Authors:** Kohei Umezu, Kenshiro Hara, Yuuki Hiradate, Takashi Numabe, Kentaro Tanemura

**Affiliations:** 1 Laboratory of Animal Reproduction and Development, Graduate School of Agricultural Science, Tohoku University, Sendai-shi, Miyagi, Japan; 2 Miyagi Agricultural Development Corporation, Sendai-shi, Miyagi, Japan; School of Sciences and Languages, Sao Paulo State University (UNESP), BRAZIL

## Abstract

Sperm migration towards an oocyte in the female reproductive tract is an important step for successful fertilization. Although several sperm-chemotactic factors have been identified in mammals, it is unclear whether these chemoattractants contribute to sperm migration towards an oocyte that is the final destination for sperm. Furthermore, chemoattractants for bovine sperm are still undiscovered even though the follicular fluid attracts sperm in cattle. Here, we demonstrated that a single bovine cumulus-oocyte complex (COC) had the ability to attract sperm, suggesting that the COC secreted sperm chemoattractants. We identified stromal cell-derived factor 1 (SDF1), which was expressed in COCs, and its receptor CXCR4 in sperm, as a candidate. Our results showed that bovine sperm preferentially migrated to the area with a high SDF1 concentration and occasionally showed turn movements by asymmetric flagellar bends during the migration. We also demonstrated that increasing the intracellular Ca^2+^ concentration via Ca^2+^ channels was related to SDF1-induced sperm chemotaxis. Finally, a CXCR4 inhibitor significantly suppressed the *in vitro* bovine sperm migration towards a COC. Taken together, we propose that SDF1 is a chemotactic factor for bovine sperm to regulate their migration towards an oocyte via the CXCR4 receptor.

## Introduction

Sperm migration towards an oocyte is the first crucial step for successful fertilization and subsequent conception. After ejaculation or artificial insemination, mammalian sperm migrate to the ampulla of an oviduct, which is the fertilization site, and very few of them succeed in entering the oviduct [1]. This limited number of sperm still has to swim a long distance and overcome several obstacles to reach an oocyte in the ampulla of the oviduct. This fact and the very small dimension of the cumulus-oocyte complex (COC) relative to that of the oviduct indicate the need for sperm guidance mechanisms [2]. Sperm chemotaxis is a prevalent phenomenon from marine invertebrates with external fertilization to mammals with internal fertilization and is considered to assist sperm migration as well as thermotaxis, rheotaxis, and muscle contractions in the female reproductive tract [3]. Because thermotaxis, rheotaxis, and muscle contractions are all long-range mechanisms to guide sperm to the upper part of the female reproductive tract [2], chemotaxis is only a guidance mechanism to modulate the swimming direction precisely just prior to reaching an oocyte.

Several sperm-chemotactic factors have been identified in humans and mice [4–7]. However, it is unclear whether these chemotactic factors contribute to the migration towards an oocyte that is the final destination for sperm. In fact, one study has suggested that oocytes and cumulus cells secrete sperm chemotactic factors and attract sperm in humans [8], whereas another study indicated that a COC is incapable of attracting sperm in mice [5]. The causes of these contradictory findings remain obscure, but it might result from interspecies differences, particularly the difference between monotocous and polytocous species. In monotocous species such as humans and cattle, sperm chemotaxis is more reasonable because finding a sole oocyte in the extensive tract is more difficult, and the chemical gradient is easier to create because there are no other origins of sperm chemoattractants. Accordingly, we hypothesized that chemotactic factors from an oocyte assist sperm migration in monotocous species. To test this hypothesis, we focused on cattle because chemotactic factors for bovine sperm are still undetermined and aimed to identify a chemotactic factor that leads sperm to an oocyte.

Chemokines have the ability to attract cells in response to a chemical gradient of the stimulus and are involved in several biological events, especially those related to the immune response [9]. Stromal cell-derived factor 1 (SDF1; also known as CXCL12) is a member of the CXC family of chemokines, and a specific ligand for chemokine CXC motif receptor 4 (CXCR4). SDF1-CXCR4 signaling plays an important role in chemotaxis of several cell types such as lymphocytes [10], megakaryocytes [11], and primordial germ cells [12, 13]. They also participate in several *in vivo* events such as tumor metastasis [14], joint infiltration [15], and human immunodeficiency virus-1 infection [16].

Some studies have suggested that follicular fluid attracts bovine sperm [17, 18]. Progesterone exists in mammalian follicular fluids and a well-known chemoattractant [19]. However, one study has showed that progesterone is not the major chemoattractant in follicular fluid in humans, suggesting the existence of other chemotactic factors in follicular fluid. Recently, SDF1 was also reported to be expressed in human follicular fluid, and *in vitro* experiments have shown that SDF1 has the ability to attract human sperm [20]. Therefore, we focused on SDF1 as a candidate chemotactic factor in cattle as well as humans. Overall, the objective of this study was to investigate the possibility that SDF1 leads sperm to an oocyte in cattle and to examine whether/how SDF1-CXCR4 signaling regulates *in vitro* sperm migration towards an oocyte.

## Materials and methods

### Ethics statement

All of the animal care and experiments of this study were conducted in compliance with the Regulations for Animal Experiments and Related Activities at Tohoku University. The study was approved by the Tohoku University Institutional Animal Care and Use Committee (2016-048-2, 2019-003-1).

### Sperm and cumulus-oocyte complex preparations

Bovine sperm samples were prepared as reported previously [21]. Briefly, we used four semen straws from different mature Japanese Black cattle bulls in this study. Four of the bulls had known fertility and each semen sample contained at least 50% progressively motile sperm after thawing. Each frozen semen sample was thawed in a water bath at 38.5°C for 15 sec and then transferred to 1.5 mL capped plastic tubes with 1 ml phosphate-buffered saline (PBS, pH 7.4). After centrifugation at 430 × *g* for 5 min at room temperature, the supernatant was discarded and the cells were resuspended in bovine gamete medium 1 (BGM-1) at 1×10^7^ cells/mL [22]. Each sperm suspension was used for each of the performed experiments.

Oocytes and cumulus cells were prepared as reported previously [23]. Ovaries were collected from Japanese Black cattle cows or heifers at a local slaughterhouse, transported to the laboratory within 4 h of removal, and placed in saline warmed to 38.5°C. Follicular fluid and bovine oocytes were aspirated from antral follicles (diameter: 2–8 mm) with a 10-ml syringe attached to an 18 G needle. After the follicle contents including oocytes were precipitated, the supernatant was discarded and the cell pellet was resuspended in Medium 199 (Cat. No. 12340–030, Thermo Fisher Scientific, Waltham, MA, USA) [24]. After 10 min, the supernatant was discarded and the cell pellet was resuspended in Medium 199 again. Cumulus-oocyte complexes (COCs) with uniform ooplasm were selected in Medium 199. To compare SDF1 expression levels between immature or mature oocytes and cumulus cells, the COCs were incubated for 0, 8, or 24 h in modified Medium 199 named IVMD101 medium (Cat. No. IFP9641, Research Institute for the Functional Peptides Co., Yamagata, Japan) at 38.5°C with 5% CO_2_. After *in vitro* maturation (IVM) for 0, 8, or 24 h, the COCs were washed in PBS containing 0.1% polyvinyl alcohol (PVA; Sigma-Aldrich, St. Louis, MO, U.S.A.) and then subjected to immunocytochemistry and RT-PCR. COCs matured for 24 h were also used for sperm migration assays in 12-well dishes and *in vitro* fertilization.

### *In vitro* sperm migration assay using 12-well dishes

**Fig 1:**
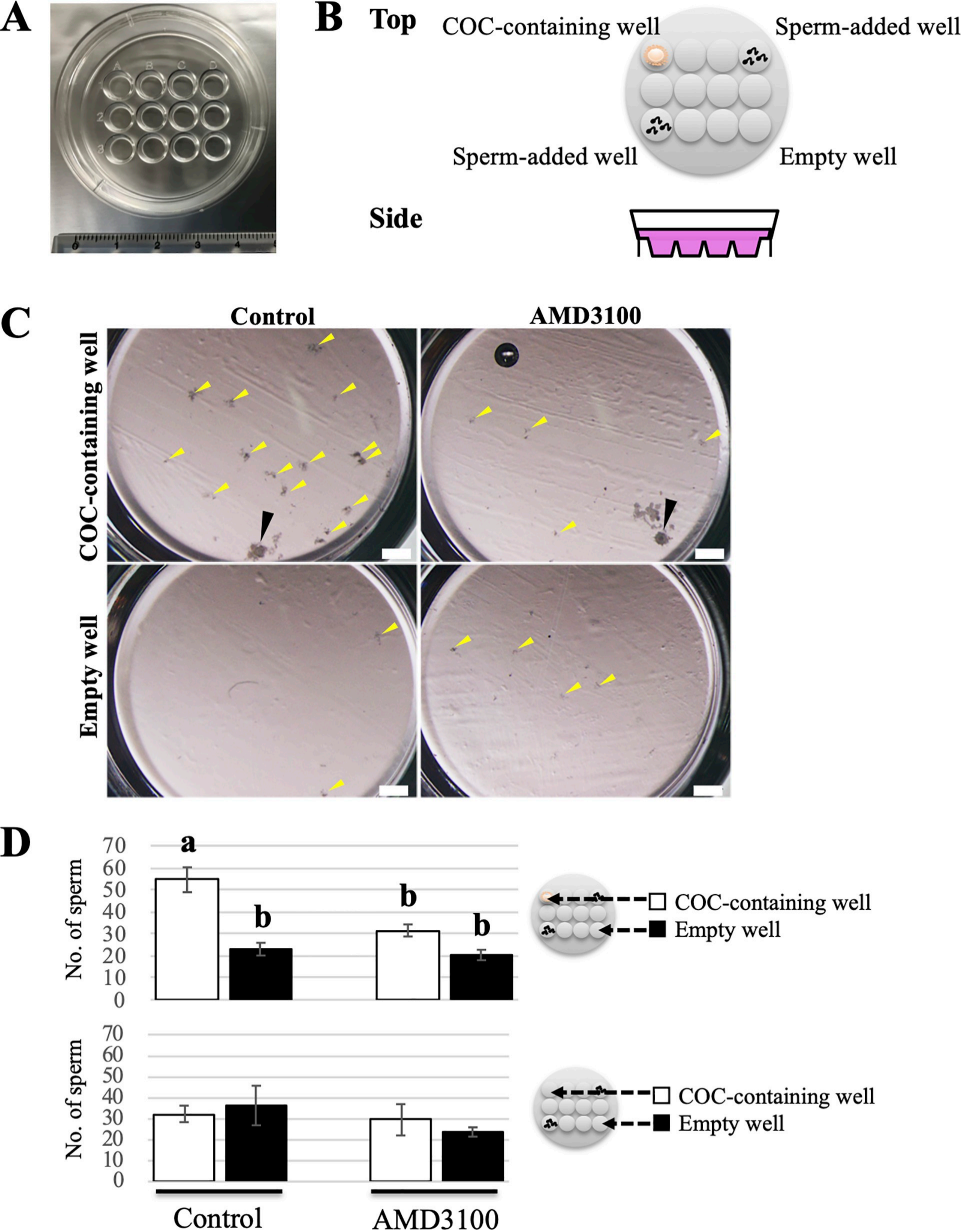
Evaluation of sperm migration toward a COC using 12-well dishes. (A) Image of a 12-well dish for the sperm migration assay. (B) Schematic illustration with both top and side views of a 12-well dish arranging a single COC and sperm. Note that the 12-well dishes were filled with medium and a single mature COC and 50 μl of sperm suspension were deliberately placed in the specified wells of the dishes as shown in this illustration so that sperm were free to swim around in the dish and go back and forth between adjacent wells. These cells were cultured for 3 h under mineral oil at 38.5°C with 5% CO_2_. (C) Representative images of COC-containing and empty wells in the presence or absence of AMD3100 after coincubation for 3 h. Yellow and black arrowheads indicate sperm aggregation and a single COC, respectively. Bars = 500 μm. (D) Comparison of the number of sperm that migrated to COC-containing and empty wells in the presence or absence of AMD3100 after three hours of incubation. Schematic illustrations on the right of each graph indicate the experimental design of the 12-well dishes. White bars show the numbers for the COC-containing well, and black bars show those for the empty well. The number of oocytes examined in this experiment is 28 for control group and 21 for AMD3100 group. Data are shown as the mean ± SE. Different letters indicate a significant difference (p<0.05). This experiment was repeated six times.

To determine whether the COCs secreted a chemoattractant for bovine sperm, we conducted *in vitro* sperm migration assays towards a COC using 12-well dishes (ART Culture Dish 12; NIPRO, Osaka, Japan). The 12-well dishes were filled with 1.5 ml modified Brackett and Oliphant’s (BO) medium named IVF100 medium (Cat. No. IFP9630, Research Institute for the Functional Peptides Co.) [25], so that sperm were free to swim around in the dish and go back and forth between adjacent wells. Then, a single mature COC and 50 μl of sperm suspension (1×10^7^ cells/mL) were deliberately placed in specified wells of the dishes (Fig 1B). These cells were cultured under mineral oil (Nacalai Tesque, Kyoto, Japan) at 38.5°C with 5% CO_2_. At three hours after the initiation of culture, 3 μl of the medium was collected from COC-containing and empty wells, and then smeared onto glass slides and dried in air for a sperm count. After rinsing with PBS, each sample was stained with Hoechst 33342 (1:1000; Thermo Fisher Scientific) for 1 h to visualize sperm cells. The treated samples were washed with PBS and covered with glass coverslips. Images from random fields (size: 1085 × 1500 μm) were obtained under a fluorescence microscope (BZ-X; KEYENCE, Osaka, Japan). Sperm migration toward a COC was evaluated by counting sperm in each of 10 fields of vision and comparing the total sperm number of COC-containing and empty wells. In the experiment to clarify whether SDF1-induced chemotaxis contributes to sperm migration towards a COC, we added the CXCR4 inhibitor AMD3100 (ab120718; abcam, Cambridge, UK) to the sperm suspension at a final concentration of 20 μM and conducted the assay in the same manner. Six hours after the initiation of the coincubation, the COCs were collected to evaluate the fertilization rate.

### *In vitro* fertilization (IVF)

IVF was performed using one straw of frozen semen and mature COCs to examine the effect of the CXCR4 inhibitor on the fertilization outcome. Sperm were resuspended in IVF100 medium at 1×10^7^ cells/mL. In some experiments, 20 μM AMD3100 dissolved in PBS was added to the sperm suspension to investigate its effect on fertilization rates. The sperm suspension was placed under paraffin oil and incubated at 38.5°C with 5% CO_2_. After washing with IVF100 medium, groups of *in vitro*-matured COCs (≤50) were introduced into 200 μl microdrops of sperm suspension in a 35-mm culture dish.

Six hours after the initiation of IVF or coincubation using the 12-well dishes, the cumulus cells from COCs were removed with a pipette, and the denuded oocytes were cultured in modified IVMD101 medium (Cat. No. IFP9651, Research Institute for the Functional Peptides Co.) at 38.5°C in an atmosphere containing 5% CO_2_, 5% O_2_, and 90% N_2_. Assessment of fertilization rates was performed as reported previously [26]. Briefly, at 18 h after IVF, the oocytes were fixed with acetic acid/ethanol (1:3). The pronuclei of each oocyte were observed and counted under a stereo microscope (Olympus, Tokyo, Japan). An oocyte with two or more pronuclei was regarded as a fertilized oocyte, while an oocyte with no or one pronucleus was regarded as an unfertilized oocyte. The total number of examined oocytes is 279 for control group of conventional IVF, 196 for AMD3100 group of conventional IVF, 33 for control group of 12-well dish IVF, and 32 for AMD3100 group of 12-well dish IVF. The conventional IVF and 12-well IVF were repeated seven times and nine times, respectively.

### Western blotting and immunocytochemistry

Western blot analysis was performed to determine the expression of CXCR4 in bovine sperm as reported previously [23]. Sperm pellets were homogenized in ice-cold RIPA buffer (50 mM Tris-HCl, pH 7.6, 150 mM NaCl, 1% Nonidet P-40, 0.5% sodium deoxycholate, and 1% protease inhibitor) (Nacalai Tesque) and sonicated on ice to extract total cellular proteins. Sperm suspensions were centrifuged, and the supernatants were collected. After the samples were resuspended in the same volume of 2× sample buffer (Nacalai Tesque), the extracted proteins were boiled for 5 min. The protein concentration was determined using the bicinchoninic acid assay kit (Nacalai Tesque). The proteins extracted from sperm (10 μg) were separated by 10% SDS-PAGE and then transferred to a polyvinylidene difluoride membrane. The membrane was blocked with Blocking One (Nacalai Tesque) for 60 min at room temperature. After three washes with PBS containing 0.1% Tween 20 (PBST), the membrane was incubated with a rabbit monoclonal anti-CXCR4 antibody (ab124824, 1:500; abcam) overnight at 4°C and then washed three times with PBST. Then, the membrane was treated with horseradish peroxidase -conjugated anti-rabbit immunoglobulin G (1:500; Promega, Madison, WI, USA) for 2 hours at room temperature. After three more washes, the membrane was reacted with Chemilumi-One (Nacalai Tesque), and images were obtained using a LAS-3000-mini Lumino Image Analyzer (Fujifilm, Tokyo, Japan).

Immunocytochemical analysis was performed to investigate the localization of CXCR4 in bovine sperm. Sperm samples were collected by centrifugation at 430 × *g* for 5 min and fixed with 2% paraformaldehyde in PBS for 30 min at 4°C. They were then washed three times with PBS and blocked with Blocking One for 60 min. Next, the sperm suspensions were incubated with the anti-CXCR4 antibody (1:100) overnight at 4°C. After three washes with PBS, the suspensions were incubated for 120 min at room temperature with Alexa Fluor 555-conjugated anti-rabbit immunoglobulin G (1:500; Thermo Fisher Scientific), Hoechst 33342 (1:5000), and FITC-conjugated peanut agglutinin lectin (PNA; 1:1000) (J Oil Mills, Tokyo, Japan). The samples were then washed, resuspended in PBS, mounted on glass slides, and then covered with glass coverslips. Images were obtained under an LSM-710 confocal laser microscope (Carl Zeiss, Jena, Germany). The rate of CXCR4-positive sperm was calculated as the number of CXCR4-positive sperm cells per total counted sperm. In the experiment to examine the effect of sperm capacitation on the CXCR4-positive sperm rate, sperm capacitation was induced by heparin treatment [27]. Briefly, sperm samples were incubated in BGM-1 medium and 10 μg/mL heparin was added for 4 hours at 38.5°C with 5% CO_2_. After incubation, immunocytochemical analysis was performed in the same manner. At least 150 sperm cells from each sample were counted.

Localization of SDF1 in bovine COCs was also evaluated by immunocytochemistry as reported previously [23]. Briefly, COCs were fixed with 2% paraformaldehyde and 1% Triton-X (Nacalai Tesque) in PBS for 1 h at 4°C. After washing with 0.1% PBS-PVA, they were blocked with 1% bovine serum albumin (Nacalai Tesque) in 0.1% PBS-PVA for 60 min. Next, the COCs were incubated with an anti-SDF1 antibody (ab155090, 1:100; abcam) overnight at 4°C. After three washes with PBS-PVA, they were incubated for 2 h at room temperature with Alexa Fluor 488-conjugated anti-rabbit immunoglobulin G (1:500) and Hoechst 33342 (1:5000). The samples were then washed, resuspended in PBS-PVA, mounted on glass slides, and covered with glass coverslips. Images were obtained under the confocal laser scanning microscope.

### Quantitative RT-PCR

Quantitative RT-PCR was performed based on our previous reports to determine how the SDF1 expression changes during the COC’s maturation process [23, 28]. Total RNA from oocytes and cumulus cells was extracted using an RNeasy micro kit (Qiagen, Venlo, Netherlands), following the manufacturer’s instructions. Oocytes and cumulus cells were separated by pipetting in PBS-PVA containing 0.1% hyaluronidase. After washing with PBS-PVA, each sample was lysed in 350 μL of RLT buffer, frozen in liquid nitrogen, and then stored at -80°C until RNA extraction. Total cell lysates were transferred to a gDNA Eliminator Spin Column (Qiagen) and centrifuged at 8,000 × *g* for 1 min. After the column was discarded, 350 μL of 80% ethanol was added to the samples. The samples were then transferred to an RNeasy MiniElute Spin Column (Qiagen), where RNA bound to the column and contaminants were washed away during subsequent wash steps with the RNA wash buffer (RW1) and the RNA ethanol-based buffer (RPE). After centrifugation at 8,000 × *g* for 1min, the spin column was placed into a new 1.5-mL tube, followed by addition of 700 μL of RW1 buffer and centrifugation at 8,000 × *g* for 1 min. The filtrate was then discarded and 500 μL RPE buffer was added to the column, followed by centrifugation at 8,000 × *g* for 2 min. The filtrate was then discarded. The column was finally placed in a 1.5-mL tube, and 15 μL of RNAse free water was added directly to the center of the column membrane, followed by centrifugation at 8,000 × *g* for 1 min for RNA elution. The total RNA was reverse transcribed to cDNA using ReverTraAce (TOYOBO, Osaka, Japan), following the manufacturer’s protocol. An RNA sample (11 μL) was converted to cDNA using1 μL of ReverTraAce, 4 μL of 5× reverse transcriptase buffer, 1 μL of random primer, 1 μL of RNase inhibitor, and 2 μL of dNTP mix. The thermocycling conditions for cDNA synthesis were as follows: 30°C for 10 min, 42°C for 20 min, and 99°C for 5 min. Samples were stored at -20°C until use in RT-qPCR analysis. Specific primers were designed based on the sequences of SDF1 (forward: CAACACTCCAAACTGCTCCC; reverse: TCGGGTCAATGCACACTTGC) and β-actin (forward: CATCGGCAATGAGCGGTTC; reverse: ACAGCACCGTGTTGGCGTAG) as an internal control. The PCR program consisted of 45 cycles of denaturation at 95°C for 5 sec, annealing at 60°C for 10 sec, and extension at 72°C for 20 sec. The obtained products were loaded onto 2% agarose gels and electrophoresed in order to confirm amplification of the correct gene. SDF1 mRNA expression levels were normalized to the β-actin mRNA level. The mean sample and endogenous control threshold cycles (Ct) for each sample were calculated using the 2-ΔΔCt method.

### Sperm migration assay using the chemotaxis chamber

To determine whether SDF1 is a chemotactic factor for bovine sperm, we evaluated sperm chemotaxis by a modified chemotaxis chamber technique (CytoSelect 96-well Cell Migration Assay, Cell Biolabs, San Diego, CA, USA) [29]. The chemotaxis chamber was separated into upper and lower chambers by a filter membrane with 8 μm pores. The lower chamber was filled with 150 μl of a sperm suspension containing about 1 × 10^7^ cells/ml in BGM-1 medium containing 10 μM caffeine. Subsequently, the upper chamber was filled with 100 μl BGM-1 medium containing 10 μM caffeine and SDF1 (ab202786; abcam) at final concentrations of 0 (Control), 0.1, 1 and 10 ng/ml. In some experiments to examine the effects of CXCR4 or Ca^2+^ channel inhibition on sperm chemotaxis, AMD3100 or NNC 55–0396 dihydrochloride (NNC; ab120265, abcam) was added to both chambers at final concentrations of 20 and 10 μM, respectively. The chamber was then incubated at room temperature for 30 min to allow for migration across the porous membrane. After incubation, the medium containing the migrated sperm in the upper chamber was collected and stained with Lysis buffer/CyQuant GR Fluorescent Dye solution at room temperature for 20 min. Fluorescence intensity was determined by a fluorescence plate reader (Beckman Coulter DTX 880 Multimode Detector, Analytical Instrument Brokers LLC, MN, USA). The sperm number was quantified based on the fluorescence intensity as described in the assay protocol.

### Sperm motility assay

Sperm motility assay was performed as reported previously to determine whether SDF1 is a sperm motility enhancer. [21]. Briefly, after equal volumes of the sperm suspension in BGM-1 medium containing 10 μM of caffeine were divided into microtubes, and SDF1, AMD3100 and/or NNC were added to each suspension at final concentrations of 0.1, 1, and 10 ng/ml, and 20 and 10 μM, respectively. After 1, 30, and 60 min, 4 μL of the samples was placed onto a 2-chamber slide with a depth of 12 μm (SC 12-01-C; Leja, Nieuw-Vennep, Netherlands). At least 100 sperm cells in five fields of a chamber were divided into motile and dead sperm, and both the percentage of motile sperm and sperm motility parameters were evaluated using a computer-assisted sperm analysis (CASA) system (SMAS, DITECT, Tokyo, Japan). Video was acquired for 1 sec at an interval of once per 1/60 sec. The evaluated sperm motility parameters were straight-line velocity (VSL, μm/sec), curvilinear velocity (VCL, μm/sec), linearity (LIN = VSL/VCL×100, %), amplitude of lateral head displacement (ALH, μm), and beat cross frequency (BCF, Hz). The trajectories of sperm were automatically extracted from the movie and overlaid on the last frame by the CASA system.

### Live imaging of sperm migration

To clarify how sperm migrated to the area with a higher concentration of SDF1, live imaging of sperm migration under a SDF1 gradient was performed by a chemotaxis slide assay as reported previously [30, 31]. The chemotaxis slides, which are commercially available, consisted of two reservoirs connected by a narrow observation area (Fig 4A) (μ-Slide Chemotaxis, ibidi, Bavaria, Germany). A time-stable chemical gradient was established by filling the left reservoirs with BGM-1 medium, and the right reservoirs were filled with BGM-1 medium containing 1 ng/ml SDF1. The presence of a time-stable chemical gradient allowed long-term observation of cellular migratory behavior. A sperm suspension of about 1 × 10^7^ cells/ml was added to the observation area under the SDF1 gradient. Sperm movement was recorded by video for 5 min under an inverted microscope (BZ-X, KEYENCE). To analyse chemotactic behaviors of sperm, we defined a turn movement as a sperm movement with more than a 180° turn in the opposite direction. During the observation for 5 min, migrated sperm were classified into two groups: sperm that migrated to the left reservoir (low SDF1 concentration) or the right reservoir (high SDF1 concentration). The turn movement rate of each group was calculated as the number of sperm with a turn movement per total migrated sperm.

### Quantification of intracellular calcium ions in sperm

To analyze the relationships between intracellular Ca^2+^ concentrations and SDF1-induced chemotaxis, the intracellular Ca^2+^ concentration of bovine sperm was measured using a commercially available kit (Cat. No. ENZ-51016, FluoForte Calcium Assay Kit, Enzo Life Sciences, Farmingdale, New York, USA), according to the manufacturer’s instructions. Before the sperm migration assay using the chemotaxis chamber, a sperm suspension was incubated with FluoForte Dye-Loading Solution for 45 min at 37°C. They were then added to the lower chamber, and BGM-1 medium containing 0 (Control) or 1 ng/ml SDF1 was added to the upper chamber. Thirty minutes after incubation, the sperm suspension was collected from the lower chamber. The fluorescence of each sample was measured using a fluorescence plate reader at Ex = 485 nm/Em 535 nm.

### TUNEL assay

Sperm DNA fragmentation was evaluated by TUNEL assay using a commercially available kit (Cat. No. C10617, Click-iT^™^ Plus TUNEL Assay, Thermo Fisher Scientific) to compare the DNA quality between SDF1-attracted and unattracted sperm. After the sperm migration assay using the chemotaxis chamber as described above, we collected the medium containing sperm from both the upper and lower chambers. The samples were fixed with 2% paraformaldehyde and 0.1% Triton X-100 in PBS for 30 min at 4°C. They were then washed three times with PBS, smeared onto glass slides, and dried in air. After washing with PBST three times, TdT reaction buffer was added to each slide, followed by incubation for 60 min at 37°C, according to the manufacturer’s instructions. The samples were washed and then incubated with 50 μl TUNEL reaction buffer for 30 min at 37°C in the dark with a humidified atmosphere. They were washed three times, stained with Hoechst 33342 (1:1000) for 60 min, and then washed again. Sperm samples were observed under a fluorescence microscope. At least 100 sperm cells from each chamber were divided into two groups: TUNEL-positive and TUNEL-negative cells.

### Mitochondrial membrane potential assay by jc-1 staining

To compare the mitochondrial quality between attracted and unattracted sperm, mitochondrial membrane potential (MMP) of sperm was evaluated by 5,5′,6,6′-tetrachloro-1,1′,3,3′-tetraethylbenzimidazolylcarbocyanine iodide (JC-1) staining using a commercially available kit (Cat. No. M34152, MitoProbe^™^ JC-1 Assay Kit, Thermo Fisher Scientific). Briefly, after the sperm migration assay using the chemotaxis chamber as described above, the medium was collected from the upper and lower chambers. Sperm samples were incubated with 2 μM JC-1 for 20 min at 37°C with 5% CO_2_. After washing with BGM-1 medium, they were stained with Hoechst 33342 (1:1000) for 30 min. The samples were then washed, suspended in BGM-1 medium, mounted on glass slides, and covered with glass coverslips. Images were obtained under the fluorescence microscope. At a high MMP, JC-1 forms J-aggregates inside mitochondria and emits orange/red fluorescence, whereas in a low MMP state, it will remain in the monomer form and emit green fluorescence (Fig 5C). At least 100 sperm cells from each chamber were divided into high MMP and low MMP cells.

### Statistical analysis

All experiments were repeated at least three times. Data are presented as means ± the standard error (SE). Statistical analyses were carried out using the two-tailed Student’s t-test for a single comparison and the Tukey-Kramer test or Dunnett’s test for multiple comparisons. Fertilization, cleavage, and developmental rates were analyzed by the chi-squared test. Values of *p*<0.05 were considered to indicate significant differences (*p<0.05 and **p<0.01).

## Results

### Bovine COCs attract spermatozoa

First, an *in vitro* sperm migration assay towards a COC using 12-well dishes was performed to determine whether the COC secretes a chemoattractant for bovine sperm. The 12-well dishes were filled with medium, so that sperm were free to swim around, and a single mature COC and sperm suspension were placed in specified wells of the dishes (Fig 1A and 1B). At three hours after the initiation of culture, some sperm aggregation was observed in all wells. However, there was more sperm aggregation in COC-containing wells than in empty wells (Fig 1C, left). Based on this result, we hypothesized that bovine sperm preferentially moved to COC-containing wells rather than empty wells. To test this hypothesis, the number of migrated sperm in each well was counted after three hours of incubation. The results showed that the number of migrated sperm in COC-containing wells was significantly higher than that of migrated sperm in the empty wells (Fig 1D, top), which was in agreement with our hypothesis. Moreover, when a single COC was not placed on a COC-containing well, there were no significant differences between the sperm numbers in both wells (Fig 1D, bottom). Collectively, the *in vitro* assay using 12-well dishes demonstrated that a single bovine COC has the ability to attract sperm, suggesting that a COC secretes a sperm chemoattractant.

### SDF1 is expressed in COCs and CXCR4 is expressed in spermatozoa

**Fig 2:**
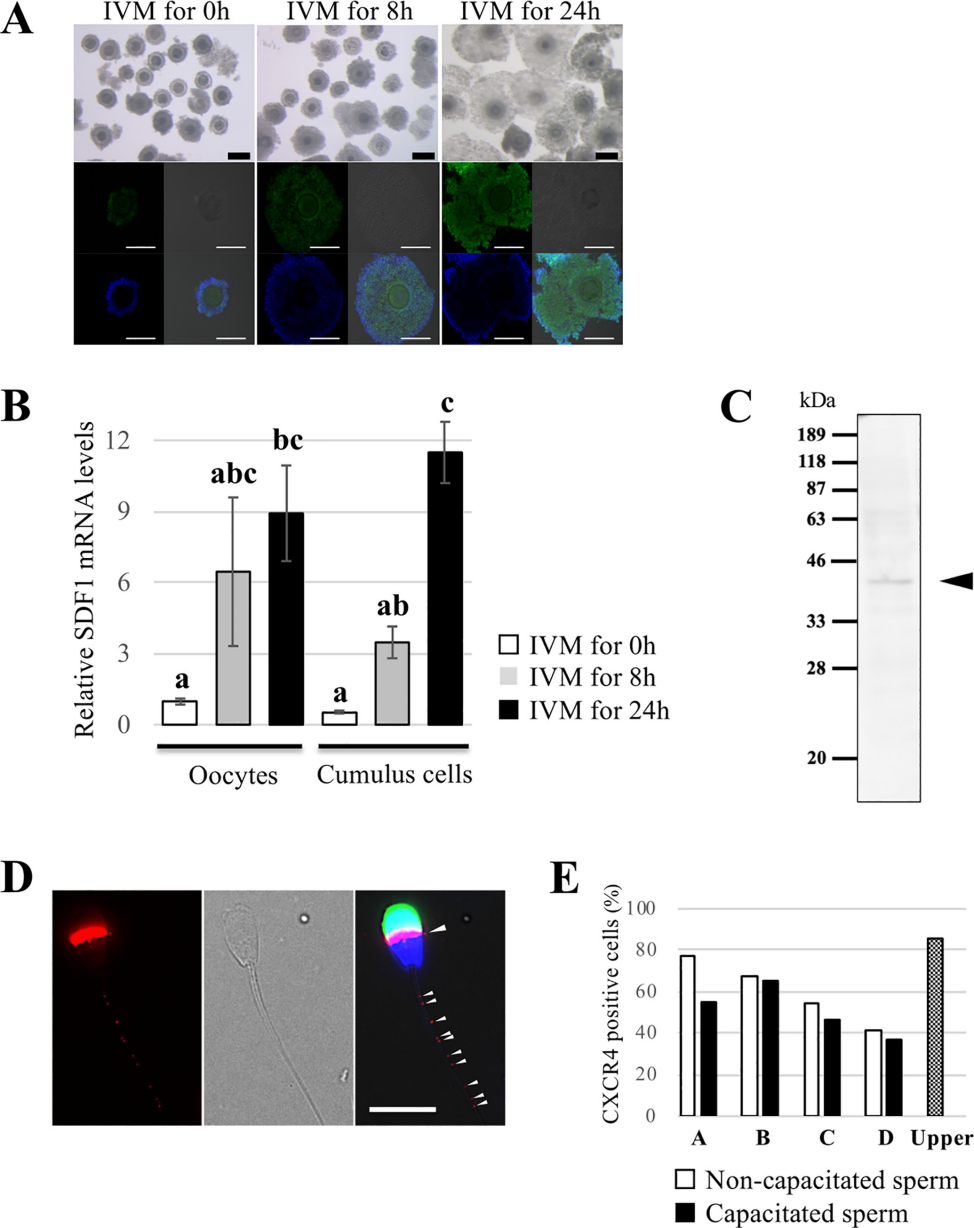
Detection of expression of CXCR4 in spermatozoa and SDF1 in COCs. (A) Representative immunocytochemical images showing SDF1 localization and brightfield images of bovine COCs. Oocytes and cumulus cells were matured *in vitro* for 0, 8, or 24 h. Note the immunoreactivity detected in the cumulus cells and oocytes, and their expression levels increased during IVM. Blue: nuclei (Hoechst 33342); Green: SDF1. Bars = 100 μm. (B) Relative mRNA expression of SDF1 in oocytes and cumulus cells was measured by quantitative RT-PCR. Relative SDF1 mRNA expression was normalized to β-actin mRNA expression. White, grey, and black bars show relative SDF1 mRNA expression after IVM for 0, 8, and 24 hours, respectively. Data are shown as the mean ± SE. n = 3. Different letters indicate a significant difference (p<0.05). (C) CXCR4 expression was detected by western blot analysis. Black arrowhead indicates the specific band corresponding to CXCR4. (D) Representative immunocytochemical image showing CXCR4 localization in bovine sperm. Spot-like immunoreactivity at the tail of sperm and immunoreactivity at the equatorial segment were detected (arrowheads). Left: Image of the CXCR4 signal (red); Middle: Brightfield image; Right: Merged image. Red: CXCR4; Blue: nuclei (Hoechst 33342); Green: PNA. Bar = 10 μm. (E) Comparison of the CXCR4-positive cells rate between four semen from different mature Japanese Black cattle bulls (A-D) with or without capacitation treatment. White bars show the rates for non-capacitated sperm, and black bars show those for capacitated sperm. The rate of sperm in the upper chamber after the chemotaxis chamber assay is also shown.

Bovine COCs were incubated and matured for 0, 8, or 24 h to compare the SDF1 expression levels of mature and immature oocytes or cumulus cells. Cumulus expansions were observed in most of the 24h IVM group and a small part of the 8h IVM group, but not in the 0h IVM group (Fig 2A, top). We performed immunocytochemical analysis using an anti-SDF1 antibody and found expression of SDF1 in COCs. Its signal intensities were also increased with the length of *in vitro* maturation time (Fig 2A, bottom). Negative control samples incubated with the primary antibody with the epitope-blocking peptide exhibited no positive staining (S1 Fig). Furthermore, we analyzed relative SDF1 mRNA expression in oocytes and cumulus cells by quantitative RT-PCR. The relative expression was increased during IVM, which was in line with the immunocytochemistry result, and there were no significant differences between relative SDF1 mRNA expression in oocytes and cumulus cells at the same time point (Fig 2B).

We next performed western blotting and immunocytochemistry using an anti-CXCR4 antibody to examine the expression and localization of CXCR4 in bovine sperm. A specific band around 39 kDa, which corresponds to the molecular size of CXCR4, was detected by western blotting (Fig 2C). Additionally, immunocytochemistry revealed CXCR4 expression in the equatorial segment and some small spots on the sperm tail (Fig 2D), which were abolished by incubation with the primary antibody preabsorbed to the blocking peptide (S2 Fig). Interestingly, about half of sperm showed no immunoreactivity for CXCR4 in this study, and there were individual differences in the rate of CXCR4-positive sperm from 41.7% to 77.2% (Fig 2E). However, there were no remarkable differences in this rate between non-capacitated and capacitated sperm.

Taken together, these findings suggest that bovine sperm have the competence to bind SDF1 secreted from mature COCs.

### SDF1 is a chemotactic factor for bovine spermatozoa

**Fig 3:**
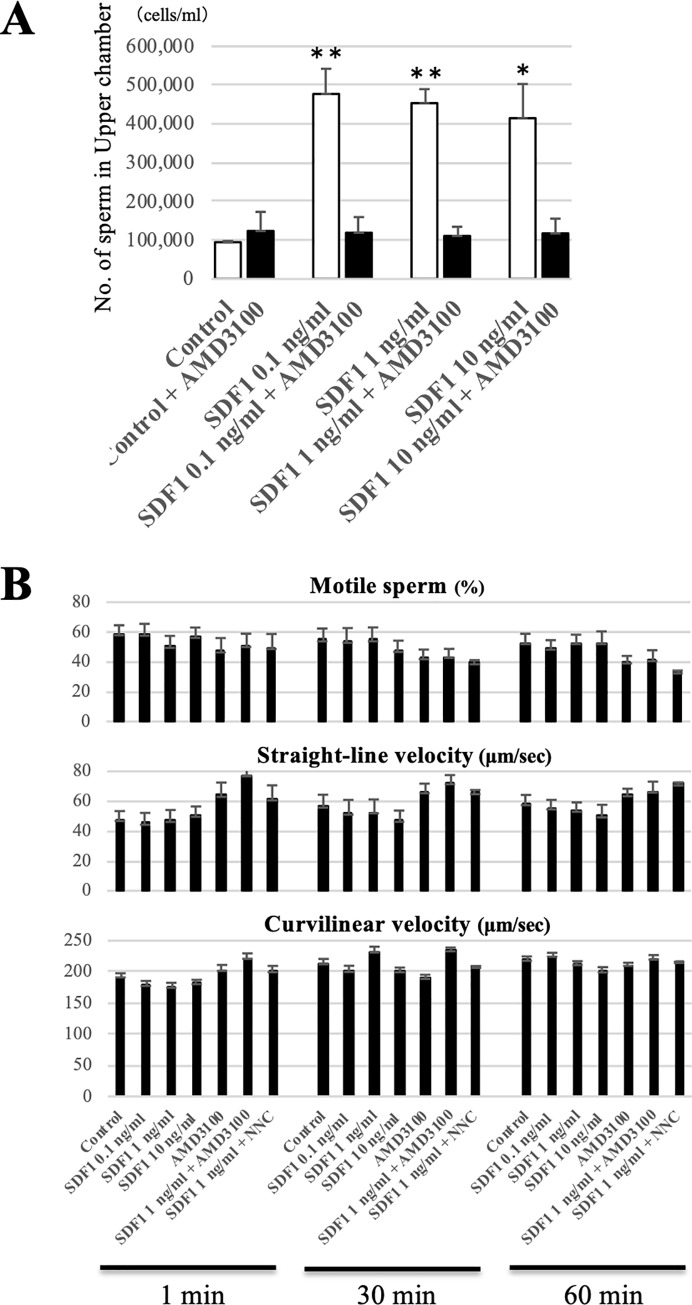
Effect of SDF1 and the CXCR4 inhibitor on *in vitro* sperm migration and motility. (A) Sperm chemotaxis toward SDF1 was evaluated by a modified chemotaxis chamber technique. The chemotaxis chamber was separated into upper and lower chambers by a filter membrane with 8 μm pores. The lower chamber was filled with a sperm suspension, and the upper chamber was filled with medium containing SDF1 at final concentrations of 0 (Control), 0.1, 1 and 10 ng/ml. The chamber was incubated for 30 min, and the number of sperm in the upper chamber was calculated. White bars show the numbers for the control group, and black bars show those for the AMD3100 group. Data are shown as the mean ± SE (*p<0.05, **p<0.01). n = 3. (B) Comparison of the motility parameters of sperm with different treatments. Each sperm sample was evaluated using a CASA system after 1, 30, and 60 min. Top: Motile (%); Middle: Straight line velocity (μm/sec); Bottom: Curvilinear velocity (μm/sec). Data are shown as the mean ± SE. n = 4.

We used the chemotaxis chamber technique to examine whether SDF1 attracts bovine sperms. The lower chamber was filled with a sperm suspension, and the upper chamber was filled with medium including 10 μM caffeine and SDF1 at final concentrations of 0 (Control), 0.1, 1 and 10 ng/ml. After incubation for 30 min, the number of sperm in the upper chamber was calculated using a plate reader. The results showed that the numbers of each SDF1 group were significantly higher than that of the control group (Fig 3A). Furthermore, addition of AMD3100, a specific CXCR4 antagonist, to each SDF1 group significantly reduced the numbers of migrated sperm. This result indicated two possibilities, namely SDF1 attracted sperm or SDF1 enhanced sperm motility. To evaluate the latter possibility, we determined the effects of SDF1 and/or AMD3100 treatment on sperm motility. Using the CASA system, we found that the average of each sperm motility parameter, including the motility rate (Motile), VSL, VCL, ALH, and BCF, was not significantly changed by any dose of SDF1 and/or AMD3100 at any time point (Fig 3B, S1 Table). This result suggests that SDF1 and/or AMD3100 do not affect bull sperm motility.

Taken together, the chemotaxis chamber and sperm motility assays suggest that SDF1 is a chemotactic factor for bovine sperm and not a sperm motility regulator.

### Turn movement, intracellular Ca^2+^, and Ca^2+^ channels are involved in SDF1-induced sperm chemotaxis

**Fig 4:**
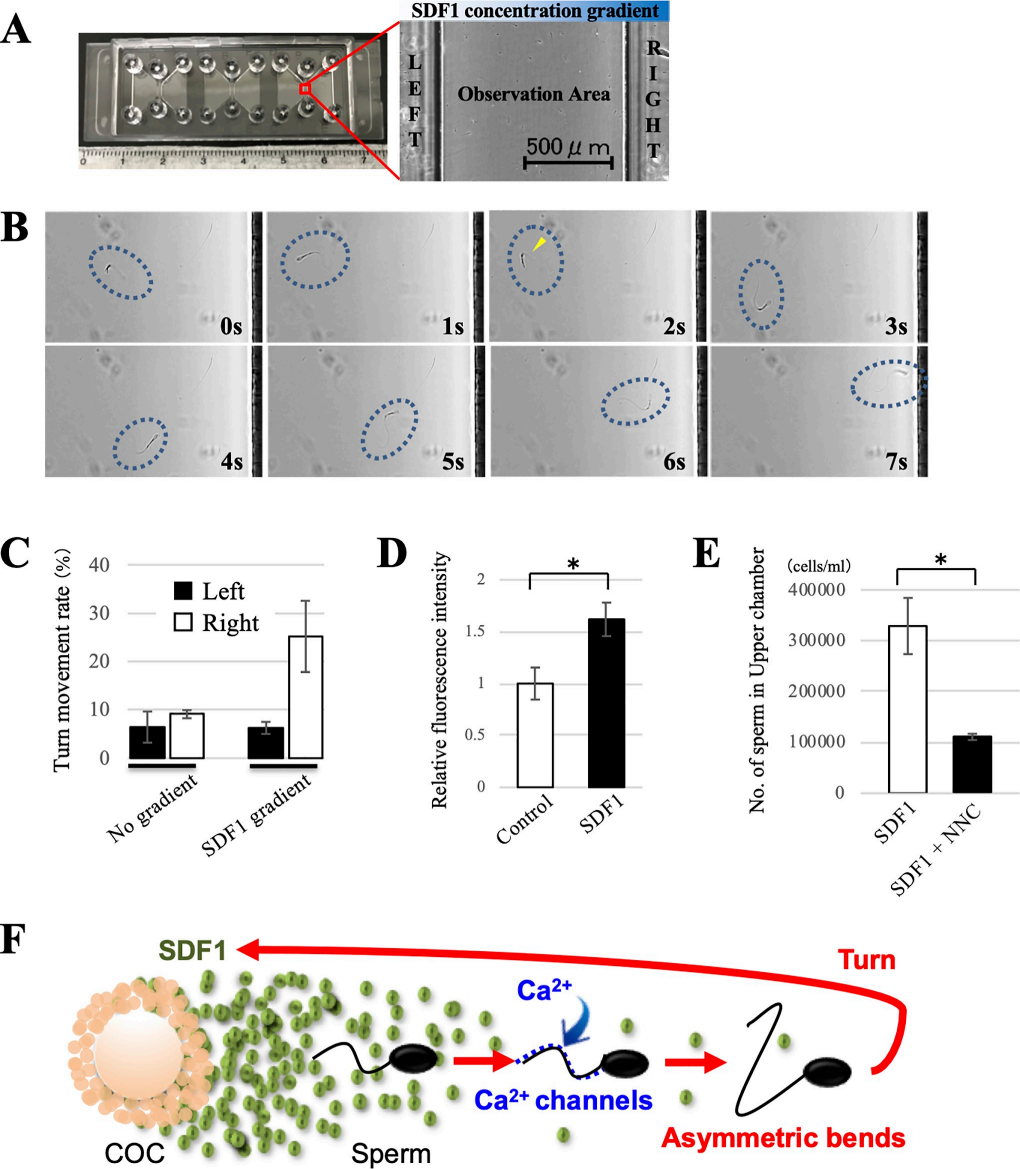
Sperm behavior during SDF1-induced chemotaxis and contribution of calcium ion. (A) Image of the chemotaxis slide consisting of two reservoirs connected by a narrow observation area. A time-stable chemical gradient was established by filling the left reservoirs with medium and the right reservoirs with medium containing 1 ng/ml SDF1. A sperm suspension was added to the observation area under a SDF1 gradient. The presence of a time-stable chemical gradient allowed long-term observation of the cellular migratory behavior. (B) Representative video images of sperm movement under a SDF1 concentration gradient with a higher concentration of SDF1 on the right. Note that the sperm showed a turn movement involving asymmetric flagellar bends (yellow arrowhead), changed their swimming direction, and migrated to the right reservoir (high SDF1 concentration). Dotted circles indicate the location of sperm. Time lapse is indicated at the lower right corner of each image. (C) The turn movement rate was evaluated to analyse the chemotactic behaviors of bovine sperm. The sperm movement was recorded by video for 5 min. A turn movement was defined as a sperm movement with a more than 180° turn in the opposite direction. The turn movement rate was calculated as the number of sperm with a turn movement per total migrated sperm. White bars show the rates for sperm that migrated to the left reservoir (low SDF1 concentration), and black bars show those for sperm that migrated to the right reservoir (high SDF1 concentrations). Data are shown as the mean ± SE. n = 3. (D) Relative intracellular Ca^2+^ levels of bovine sperm in the presence or absence of SDF1. The relative fluorescence intensity of the control group was normalised to 1. White bars show the levels for the control group, and black bars show those for the SDF1 group. Data are shown as the mean ± SE (*p<0.05). n = 6. (E) Effect of the Ca^2+^ inhibitor on sperm chemotaxis to SDF1. Sperm chemotaxis was evaluated by a chemotaxis chamber technique in the same manner. NNC was added to both chambers at a final concentration of 10 μM. White bars show the levels for the control group, and black bars show those for the NNC group. Data are shown as the mean ± SE (*p<0.05). n = 4. (F) Proposed mechanism of SDF1-induced chemotaxis for bovine sperm. The results suggest bovine sperm chemotaxis toward SDF1 is regulated as follows: (i) external Ca^2+^ uptake via Ca^2+^ channels, (ii) a rise in the intracellular Ca^2+^ concentration, (iii) asymmetric flagellar bends, and (iv) modulation of the swimming direction using the turn movement.

Live imaging of sperm migration under a SDF1 gradient was performed using chemotaxis slides to understand how bovine sperm migrated to the area with a higher concentration of SDF1. The chemotaxis slides consisted of two reservoirs connected by a narrow observation area (Fig 4A). A time-stable chemical gradient with a higher concentration of SDF1 on the right was established by filling the left reservoirs with medium, and the right reservoirs were filled with medium containing 1 ng/ml SDF1. A sperm suspension was then added to the observation area under a SDF1 gradient. During the observation for 5 min, more sperm migrated to the right reservoir (higher SDF1 concentration) rather than the left reservoir (low SDF1 concentration) (S3 Fig); this supports the data from the chemotaxis chamber assay. Moreover, sperm movement with more than a 180° turn in the opposite direction, which is defined as a turn movement, was occasionally observed during the migration (Fig 4B, S1 Movie). Detailed observation indicated that the turn movement involved asymmetric flagellar bends (Fig 4B, yellow arrowhead). To analyze the relationships between SDF1-induced chemotaxis and turn movements, we calculated the turn movement rate. Consequently, sperm migrating to the right reservoir under the SDF1 gradient showed the highest rate of turn movement compared with sperm migrating to the left reservoir or sperm under no gradient (Fig 4C). These data suggested that swimming directions of sperm migrating to the area with a high SDF1 concentration were changed by turn movements induced by asymmetric flagellar bends.

Next, we measured intracellular Ca^2+^ concentrations in sperm because intracellular Ca^2+^ plays a central role in the regulation of sperm flagellar bends. A sperm suspension was incubated with a fluorescently labelled calcium indicator before the chemotaxis chamber assay. They were added to the lower chamber, and medium with 0 or 1 ng/ml SDF1 was added to the upper chamber. Thirty minutes after incubation, we collected the sperm suspension from the lower chamber and measured the fluorescence intensity. The result demonstrated that sperm in the presence of SDF1 showed significantly a higher fluorescence intensity than sperm in the absence of SDF1, suggesting that the SDF1 gradient enhanced intracellular Ca^2+^ concentrations in sperm (Fig 4D).

We next examined the involvement of Ca^2+^ channels, which primarily control the entry of Ca^2+^ into sperm cells, in SDF1-induced chemotaxis. Before the chemotaxis chamber assay, a sperm suspension was incubated with 0 (Control) or 10 μM NNC, a highly selective T-type Ca^2+^ channel blocker. After 30 min of incubation, the chemotaxis chamber assay employing SDF1 was performed as described above. The number of sperm in the upper chamber was significantly decreased by the addition of NNC (Fig 4E). Furthermore, NNC did not have a significant effect on sperm motility parameters (Fig 3B). These results suggested that inhibition of Ca^2+^ channels suppressed SDF1-induced chemotaxis for bovine sperm.

Overall, sperm occasionally show turn movements by asymmetric flagellar bends during migration to an area with a high SDF1 concentration. Additionally, intracellular the Ca^2+^ concentration and Ca^2+^ channels of sperm are involved in SDF1-induced sperm chemotaxis.

### SDF1-attracted sperm have higher quality DNA and mitochondria than unattracted sperm

**Fig 5:**
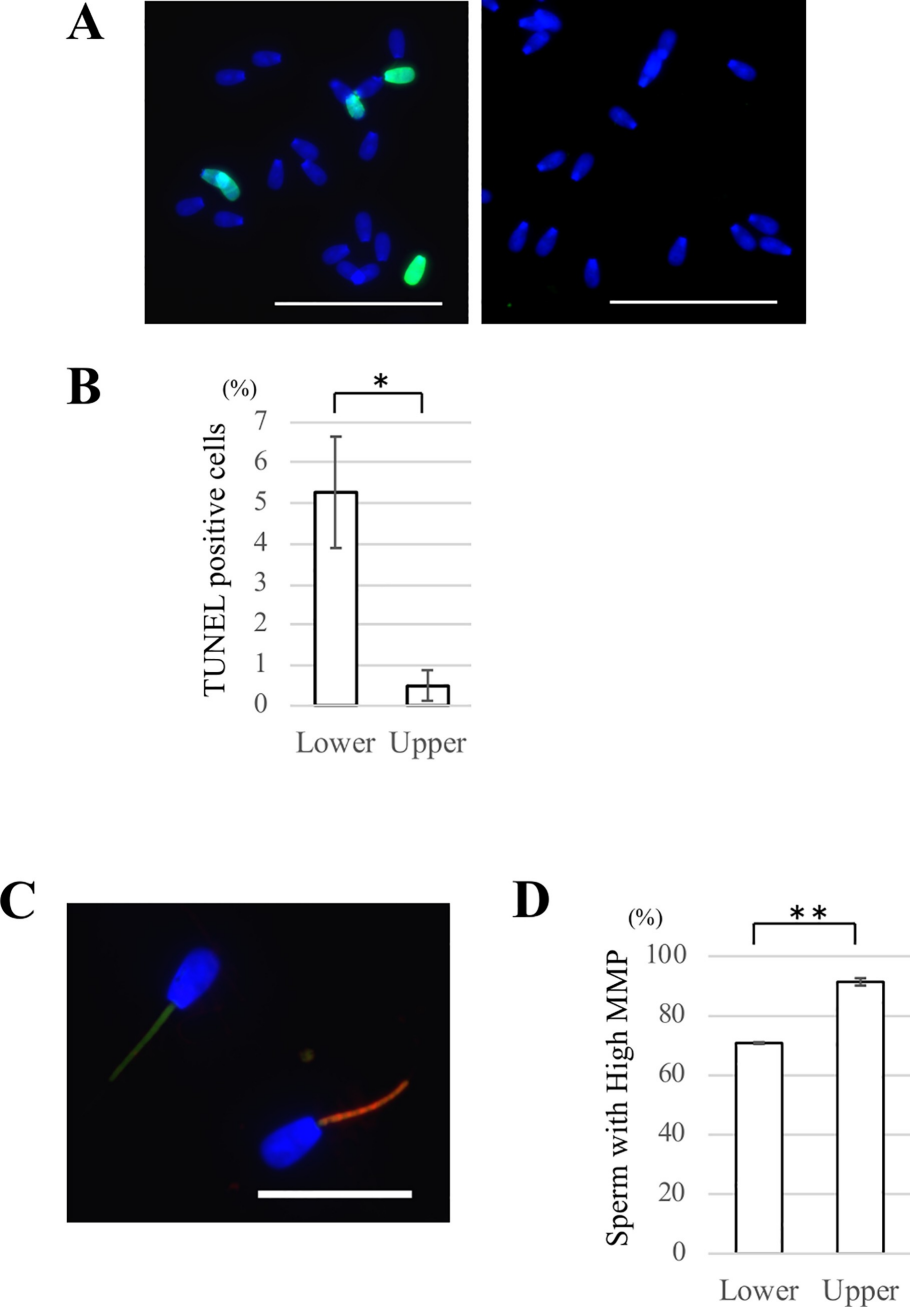
Comparison of the DNA and mitochondrial quality of attracted and unattracted sperm by SDF1. (A) Detection of sperm DNA fragmentation by TUNEL assays. After the chemotaxis chamber assay, the medium from both upper and lower chambers was collected. The samples were fixed, smeared onto glass slides, and subjected to TUNEL staining. Left: representative image of sperm in the lower chamber; Right: representative image of sperm in the upper chamber. Blue: nuclei (Hoechst 33342); Green: TUNEL-positive cells. Bars = 50 μm. (B) Comparison of the rates of TUNEL-positive sperm in lower chamber and upper chambers. At least 100 sperm cells from each chamber were divided into two groups: TUNEL-positive and TUNEL-negative cells. Data are shown as the mean ± SE (*p<0.05). n = 4. (C) Representative image of sperm cells stained in the JC-1 assay. MMP of sperm was evaluated by the JC-1 assay after the chemotaxis chamber assay. Left: sperm with green fluorescence staining representative of inactive mitochondria with a low membrane potential; Right: sperm with orange or red fluorescence staining representative of active mitochondria with a high membrane potential. Blue: nuclei (Hoechst 33342). Bar = 20 μm. (D) Comparison of the rates of of sperm with a high MMP in lower and upper chambers. At least 100 sperm cells from each chamber were divided into high and low MMP cells. Data are shown as the mean ± SE (**: p<0.01). n = 3.

Sperm DNA fragmentation was investigated by TUNEL assays to evaluate sperm DNA quality. After the sperm migration assay using the chemotaxis chamber, we collected the sperm medium from both upper and lower chambers. The sperm samples were incubated with TUNEL reaction buffer, observed under a fluorescence microscope, and divided into two groups: TUNEL-positive and TUNEL-negative cells. The rate of TUNEL-positive sperm in the upper chamber was significantly less than that in the lower chamber (Upper 0.5 ± 0.4% vs. Lower 5.3 ± 1.4%) (Fig 5A and 5B). This result indicated that sperm that had migrated to the area with high SDF1 seldom had DNA damage.

Sperm MMP was investigated by JC-1 staining because it is an indicator of the energetic state of mitochondria and identifies functional intact mitochondria related to sperm motility. After the sperm migration assay using the chemotaxis chamber, we collected the medium from the upper and lower chambers. Both samples were incubated with JC-1, observed under a fluorescence microscope, and divided into high and low MMP cells (Fig 5C). The rate of sperm with high MMP in the upper chamber was significantly higher than that in the lower chamber (Upper 91.4 ± 1.2% vs. Lower 70.8 ± 0.3%) (Fig 5D). These data suggested that there were much more sperm with functionally intact mitochondria in the upper chamber.

Taken together, sperm that had migrated to the area with SDF1 (SDF1-attracted sperm) had less DNA damage and more functionally intact mitochondria, indicating high quality of DNA and mitochondria.

### CXCR4 inhibitor negatively affects *in vitro* sperm migration towards a COC, but not fertilization

We evaluated the effect of a CXCR4 inhibitor in the sperm migration assay using 12-well dishes to determine whether the SDF1-CXCR4 signalling plays a role in leading sperm to the COC. Before performing the assay, AMD3100 was added to the sperm suspension at a final concentration of 20 μM, and then the assay was conducted in the same manner. We observed less sperm aggregation in the COC-containing well of the AMD3100 group than that of the control group (Fig 1C, top). This observation was an agreement with the result showing that the number of migrated sperm in the COC-containing well after three hours of coincubation was significantly decreased by the addition of AMD3100 (Control 54.5 ± 5.6 cells vs. AMD3100 23.0 ± 2.9 cells) (Fig 1D, top). This result suggested that CXCR4 is involved in *in vitro* sperm migration towards a COC in cattle.

**Fig 6:**
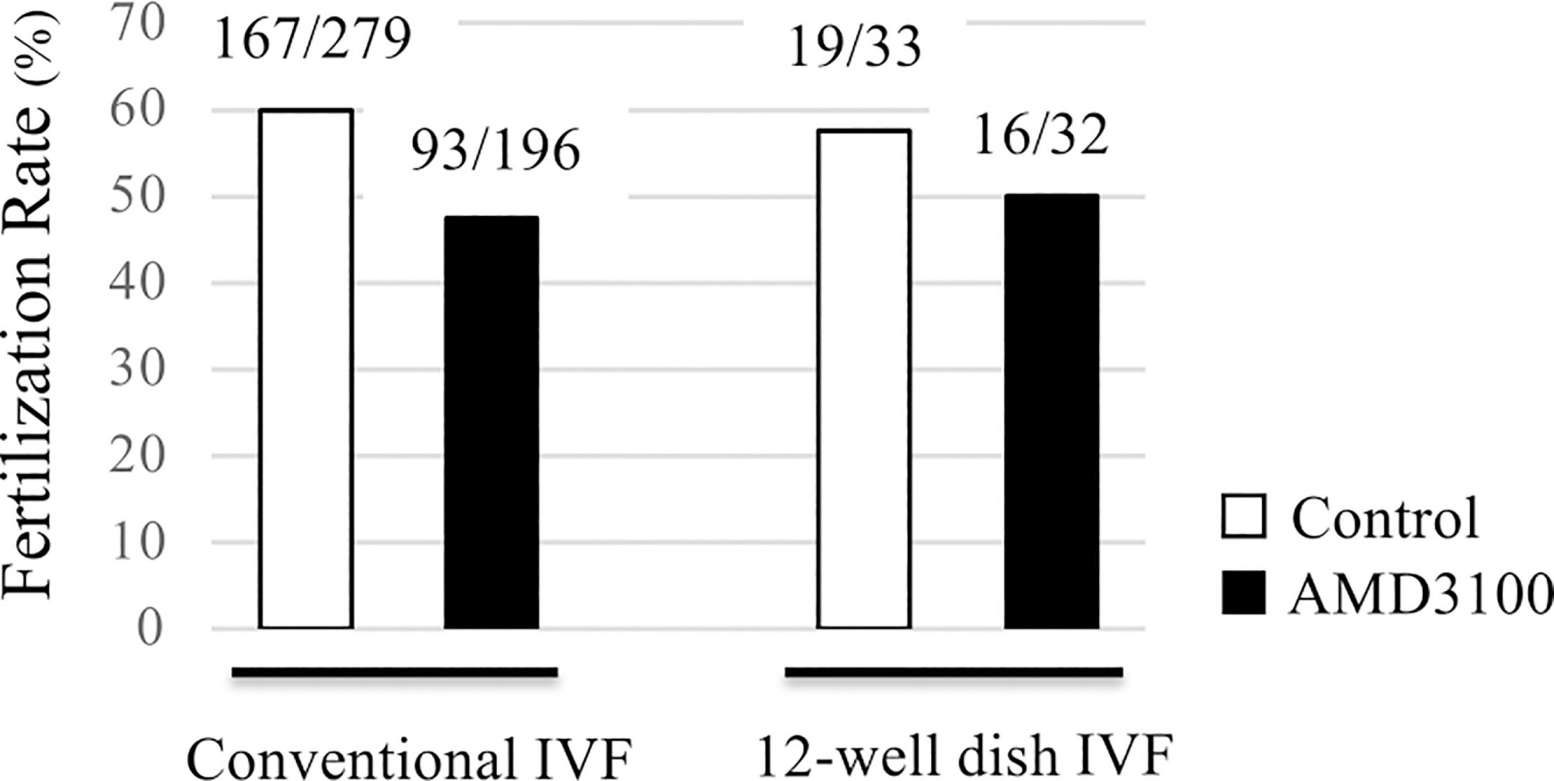
Effect of the CXCR4 inhibitor on the *in vitro* fertilization rate. The fertilization rates of conventional IVF and 12-well dish IVF in the presence or absence of AMD3100. In conventional IVF, groups of *in vitro*-matured COCs (≤50) were introduced into 200 μl microdrops of a sperm suspension in a 35-mm culture dish and cocultured for 6 h. In the 12-well dish IVF, a single mature COC and sperm suspension were placed in the specified wells of the dishes and cocultured for 6 h. Then, 20 μM AMD3100 was added to the sperm suspension to investigate its effect on fertilization rates. Eighteen hours after coincubation, denuded oocytes were fixed with acetic acid/ethanol. The pronuclei of each oocyte were observed, and an oocyte with two or more pronuclei was regarded as a fertilized oocyte. White bars show the numbers for the control group, and black bars show those for the AMD3100 group. The numbers above each bar indicate the number of fertilized oocytes per total number of evaluated oocytes.

Finally, the fertilization rates of both control and AMD3100 groups after coincubation using 12-well dishes were evaluated to examine how important the SDF1-CXCR4-induced sperm migration was for fertilization in cattle. The fertilization rate of the control group was higher than that of the AMD3100 group, but there was no significant difference (Control 57.58% vs. AMD3100 50.0%) (Fig 6). Similar results were obtained from the conventional method of IVF (Control 59.86% vs. AMD3100 47.45%). The subsequent cleavage rate at day 2 and the blastocyst formation rate at day 8 also showed no significant differences between control group and AMD3100 groups (S2 Table). These results indicated that CXCR4 inhibition did not exert a significant effect on the fertilization outcome and subsequent embryo development.

## Discussion

In this study, we first examined whether a bovine COC attracts sperm by chemotaxis. A single COC had the ability to attract sperm (Fig 1D), suggesting that a bovine COC secretes sperm chemoattractants similarly to humans [8]. In humans, SDF1 is expressed in oocytes and secreted into follicular fluid, which has a chemotactic ability for sperm [20]. Thus, we focused on SDF1 as a candidate chemotactic factor in cattle and investigated the expression of SDF1 in COCs and its receptor in sperm.

SDF1 was detected in bovine oocytes and cumulus cells, which increased during IVM (Fig 2A and 2B). Additionally, the enzyme-linked immuno-sorbent assay showed that the SDF1 concentration in bovine follicular fluid was approximately 29 ng/ml (S4 Fig), indicating that a bovine COC secretes SDF1 into its surroundings. Because only a very small fraction of follicular fluid is transported into the oviduct which is the fertilization site, we set the SDF1 concentrations as 0.1, 1, and 10 ng/ml in this study. Next, we investigated the presence of the specific SDF1 receptor CXCR4 in bovine sperm. CXCR4 expression was localized in the equatorial segment and sperm tail of about half of total sperm (Fig 2D and 2E). Although the localization of CXCR4 was different from that in humans, there was agreement that only some sperm expressed CXCR4 [20]. The differences between CXCR4-positive and CXCR4-negative sperm would not result from the capacitated state or acrosome reaction because the rate of CXCR4-positive cells was not greatly affected by *in vitro* capacitation treatment and the presence or absence of the acrosome. However, this result is contradictory to previous studies suggesting that the expression of sperm chemotaxis receptors, such as natriuretic peptide receptor 1/2 and CCR6, is enhanced by sperm capacitation treatment [4, 5, 32]. Further studies are needed to understand the biological significance of the heterogeneous expression of CXCR4 in sperm. Overall, we propose that some bovine sperm have the competence to sense SDF1 secreted from mature COCs.

Our results indicated that SDF1 is a chemotactic factor for bovine sperm and not a sperm motility regulator. It was also found that SDF1 is not a regulatory factor of sperm protein tyrosine phosphorylation, which is a major indicator of sperm capacitation, or the acrosome reaction (S5 Fig). According to our results, <5% of total sperm were attracted by SDF1, which was in agreement with previous studies suggesting that only a small fraction of the sperm population is chemotactically responsive in mammals [6, 18, 33]. In fact, the percentage of chemotactic sperm in the total sperm population is 2%–12% in humans [34]. Although the reasons why only a small percentage of sperm has a chemotactic ability are still controversial, three possibilities may explain this phenomenon. One possibility is the lack of chemotaxis receptors in some sperm as mentioned above. Several chemotaxis receptors including CXCR4 are expressed in only a small fraction of sperm, suggesting that a large proportion of the sperm population is unable to sense chemoattractants intrinsically. Another possibility is the heterogeneity of the sperm capacitated state. Several studies have demonstrated that only capacitated sperm, which possess the potential to undergo the acrosome reaction and hyperactivation, are chemotactic in humans and mice [32, 33, 35]. However, in cattle, the capacitated state is unlikely to be relevant to sperm chemotaxis because this study and a previous study showed that sperm capacitation treatment is unnecessary to induce sperm chemotaxis in cattle [18]. It should be noted that in this study, the sperm medium contained caffeine that induces hyperactivation immediately regardless of the sperm capacitated state (S6 Fig) [36]. Indeed, the number of sperm attracted by SDF1 was decreased by the absence of caffeine in the sperm medium (S7 Fig). Thus, we propose that hyperactivated sperm which are a small proportion of the population, are chemotactically active rather than capacitated sperm in cattle. The other possibility is the heterogeneity of sperm quality. In this study, the SDF1-attracted sperm had higher quality DNA and mitochondria than unattracted sperm (Fig 5). Prior studies also suggest that chemoattracted sperm have low DNA fragmentation or a higher rate of sperm with a high MMP [20, 37, 38]. In addition, the cleavage rate of oocytes fertilized with sperm selected by progesterone-induced chemotaxis is significantly higher compared with unselected sperm [38]. Therefore, these data imply that only physically optimal sperm are chemotactically responsive to ensure successful fertilization. Taken together, a plausible explanation for only a small percentage of chemoattracted sperm is the heterogeneity of the physiological state of mammalian sperm, such as the expression of receptors, capacitated state, and quality of DNA and mitochondria.

Little is known about the behavioral and molecular mechanisms of sperm chemotaxis in cattle. Chemotaxis is characterized by directional changes in the movement towards the source of the chemoattractant. Therefore, we first observed sperm movement and behavior in a SDF1 concentration gradient. As a result, bovine sperm occasionally showed turn movements by asymmetric flagellar bends during migration to the area with high SDF1 concentrations. Such turn movements during sperm migration by chemotaxis are also observed in humans and several marine invertebrate species [39–43]. Thus, the turn movement by asymmetric flagellar bends is likely to be a typical and common phenotype of sperm during chemotactic behavior. There is a widespread consensus that intracellular Ca^2+^ is the primary factor regulating flagellar waveform symmetry. Therefore, we measured intracellular Ca^2+^ concentrations in sperm in the presence or absence of SDF1 to determine whether intracellular Ca^2+^ modulates the turn movements during chemotactic behavior. Our results indicated that the SDF1 gradient enhanced intracellular Ca^2+^ concentrations of bovine sperm (Fig 4D). Increasing Ca^2+^ concentrations in sperm during chemotactic behavior has also been reported by previous studies of other species [6, 20, 44–46]. Additionally, bovine sperm did not show chemotaxis toward SDF1 when we omitted Ca^2+^ from the sperm medium (S7 Fig). These results suggest that increasing Ca^2+^ concentrations induce asymmetric flagellar bends involved in the turn movements, which play an important role in sperm chemotaxis. Finally, we examined the relationship between sperm chemotaxis and Ca^2+^ channels because these channels primarily control Ca^2+^ entry into sperm cells [47–50]. Our results showed that the Ca^2+^ channel inhibitor NNC suppressed SDF1-induced chemotaxis of bovine sperm (Fig 4E). This result agrees with findings in sea urchin, indicating that CatSper, a sperm-specific Ca^2+^ channel inhibitor, abolishes chemotaxis of sperm in a chemoattractant gradient [51, 52]. Overall, we propose that Ca^2+^ entry via Ca^2+^ channels is a critical mechanism for sperm chemotaxis. Moreover, the Ca^2+^ influx through Ca^2+^ channels induces hyperactivation [53]. In fact, the Ca^2+^ channel inhibitor NNC suppressed not only sperm chemotaxis, but also hyperactivation (S6 Fig). In addition, asymmetric flagellar bends are common characteristics of chemotaxis and hyperactivation [54]. A previous study has suggested that hyperactivation is involved in the chemotactic response of human sperm [55]. Indeed, progesterone is well known as both a hyperactivation inducer [56, 57] and chemoattractant [58,59]. Thus, the Ca^2+^ influx through Ca^2+^ channels may allow sperm to be hyperactivated and chemotactically active simultaneously, and these two motility states may closely interact with each other for successful fertilization. In summary, a possible mechanism of bovine sperm chemotaxis toward SDF1 is as follows: (i) external Ca^2+^ uptake via Ca^2+^ channels, (ii) a rise in the intracellular Ca^2+^ concentration, (iii) asymmetric flagellar bends, and (iv) modulation of the swimming direction using the turn movement (Fig 4F).

Finally, we determined whether SDF1-CXCR4-induced chemotaxis contributes to sperm migration towards a COC by the assay using 12-well dishes. The number of migrated sperm in the COC-containing well after 3 h of coincubation was decreased significantly by addition of AMD3100 (Fig 1D), suggesting that SDF1-CXCR4 signaling is involved in bovine sperm migration towards a COC. We also evaluated the fertilization outcome after coincubation using 12-well dishes, but there were no significant differences in fertilization, cleavage, or blastocyst formation rates between control and AMD3100 groups (Fig 6, S2 Table). Overall, we propose that SDF1-CXCR4-induced chemotaxis increases the frequency of contact between gametes, but does not exert a significant effect on the fertilization outcome.

Almost all previous information about sperm chemotaxis in mammals was obtained from experiments or analyses using only sperm. In contrast, this study provides new methods to evaluate sperm chemotaxis and migration toward a COC and valuable information to facilitate prediction of *in vivo* sperm chemotaxis and migration. The sperm migration assay using a 12-well dish suggested that the SDF1-induced sperm chemotaxis occurred within a few centimeters. Although sperm chemotaxis has been proposed to be a short range guidance mechanism compared with thermotaxis and rheotaxis, which are long range mechanisms, the exact range and location of the chemotaxis are unclear [2]. According to this study, mammalian chemotaxis is functionally effective in sperm migration near a COC, that is, the fertilization site. Thus, we support the idea that such chemotaxis is a short-range sperm guidance mechanism, and sperm chemoattractants act as a final modulator of sperm migration toward their proper destination.

SDF1 and other sperm chemoattractants are likely to regulate sperm migration in cattle because the CXCR4 inhibitor did not completely suppress sperm migration towards a COC. Other chemoattractants, such as progesterone as reported previously, are secreted by oocytes or cumulus cells ensure successful fertilization [58]. Detection of these other chemoattractants is needed in cattle as well as examination of the interactions and functional differences of these factors. Additionally, a further study should be conducted to determine how sperm chemotaxis, thermotaxis, and rheotaxis act together to lead sperm toward the fertilization site.

In conclusion, SDF1 is a chemotactic factor for bovine sperm to regulate their *in vitro* migration towards an oocyte via its receptor. This is the first study that has succeeded in identifying a bovine sperm chemoattractant and partially revealed the molecular and behavioral mechanism of the chemotaxis. Notably, we experimentally demonstrated that a mammalian COC utilizes the chemotaxis to ensure successful sperm migration and fertilization in a bovine model. Overall, this study provides a new insight into the process from sperm insemination to fertilization and might contribute to improving productivity in bovine reproduction.

## Supporting information

S1 Fig Negative control staining of SDF1 in COCs. Immunostaining of bovine COCs using an anti-SDF1 antibody pre-absorbed with a 10-fold excess of the epitope-blocking peptide (ab9798; abcam) overnight at 4°C. Blue: nuclei (Hoechst 33342); Green: SDF1. Bars = 200 μm.
(TIFF)

S2 Fig Negative control staining and blotting of CXCR4 in sperm. (A) Immunostaining of bovine sperm using an anti-CXCR4 antibody pre-absorbed with a 10-fold excess of the epitope blocking peptide (ab155072; abcam) overnight at 4°C. Blue: nuclei (Hoechst 33342); Green: CXCR4. Bars = 20 μm. (B) Western blotting of sperm proteins with the anti-CXCR4 antibody. Extracted sperm proteins (10 μg) were separated by 10% SDS-PAGE and transferred to a PVDF membrane. After blocking, the primary antibody diluted at 1:500 with a 10-fold excess of the blocking peptide was added as a negative control. The antibody reacted with a 39 kDa protein, which was abolished with the pre-absorbed antibody (arrowhead).
(TIFF)

S3 Fig Observation of sperm behavior in a SDF1 gradient using the chemotaxis slides. The total number of sperm migrated to the left or right reservoir during 5min of observation. White bars show the number for the sperm migrated to the left reservoir (lower SDF1 concentrations), and black bars show those for the sperm migrated to the right reservoir (higher SDF1 concentrations). Data are shown as the mean ± SE.
(TIFF)

S4 Fig SDF1 concentration in follicular fluid. SDF1 quantification in bovine follicular fluid was performed by an ELISA (Bovine Stromal Cell Derived Factor 1 ELISA Kit 96-Strip-Wells Cat. No. MBS741957; MyBiosource, Inc., San Diego, CA, USA), following the manufacturer's instructions. Follicular fluid was collected from follicles of 2–8 mm in diameter or follicles with a diameter of >8 mm of diameter. Data are shown as the mean ± SE.
(TIFF)

S5 Fig Effect of SDF1 on sperm fertilizing abilities. Sperm protein tyrosine phosphorylation, which is the major indicator of sperm capacitation, was evaluated by western blotting. Total tyrosine phosphorylation level was not significantly affected by SDF1 addition, suggesting that SDF1 don’t induce sperm capacitation in bull (S2A Fig). The effect of SDF1 on the acrosome reaction in capacitated sperm was also evaluated. After 4 hours of sperm incubation in heparin-containing BGM-1 medium, to induce capacitation, the acrosome status of sperm was determined by staining with FITC-PNA lectin. The rates of acrosome reacted sperm were not affected by SDF1 addition, suggesting that SDF1 don’t induce acrosome reaction (S2B Fig). Data are shown as the mean ± SE.
(TIFF)

S6 Fig Effects of caffeine, anti-CatSper1 antibody, and NNC 55–0396 dihydrochloride on hyperactivation. After equal volumes of the sperm suspension in BGM-1 medium were divided, the caffeine, anti-CatSper1 antibody, and/or NNC were added to each suspension at a final concentration of 10 mM, 38 μg/ml, 10 μM, respectively. The samples were placed onto 2-chamber slides with a depth of 12 μm, and observed by using an inverted microscope. At least 100 sperm of each sample were divided into motile and dead sperm, and the percentages of hyperactivated sperm per total motile sperm were calculated. Data are shown as the mean ± SE. Different letters indicate significant difference (p<0.05).
(TIFF)

S7 Fig Effects of anti-CatSper1 antibody or Ca2^+^ free medium on SDF1-induced sperm chemotaxis. Before the chemotaxis assay, sperm were incubated for 30 min with or without 38 μg/ml of anti-CatSper1 antibody. The lower chamber was filled with each sperm, and the upper chamber was filled with the medium supplemented with 1 ng/ml SDF1. The chamber was incubated for 30 min, and the number of sperm in the upper chamber was calculated. As for Ca^2+^ free medium, we omitted Ca^2+^ from the both lower and upper chamber, and conducted the assay in the same way. Data are shown as the mean ± SE.
(TIFF)

S1 Table Effects of several treatments used on bovine sperm motility parameters. VSL, straight-line velocity; VCL, curvilinear velocity (μm/sec); LIN, linearity; ALH, amplitude of lateral head displacement; BCF, beat-cross frequency. Data are shown as the mean ± SE.
(TIFF)

S2 Table Effect of AMD3100 on cleavage rate and blastocyst formation rate *in vitro*. Cleavage rate and blastocyst formation rate were assessed at 72 h and 192 h, respectively, after IVF. Cleavage rates and blastocyst formation rates were based on the number of original oocyte number and cleaved oocyte number, respectively.
(TIFF)

S1 Movie Representative of the turn movement by asymmetric flagellar bends in a SDF1 gradient. The chemotaxis chamber established a SDF1 concentration gradient with a higher concentration of SDF1 on the right than on the left.
(MOV)

S1 Raw images (PDF)

S2 Raw images (TIFF)

S3 Raw images (TIFF)
