# Supplementary figures and images for: Stromal cell-derived factor 1 regulates in vitro sperm migration towards the cumulus-oocyte complex in cattle
Source: PLoS One. 2020 Apr 30;15(4):e0232536. doi: 10.1371/journal.pone.0232536 (PMC7192438; doi:10.1371/journal.pone.0232536)

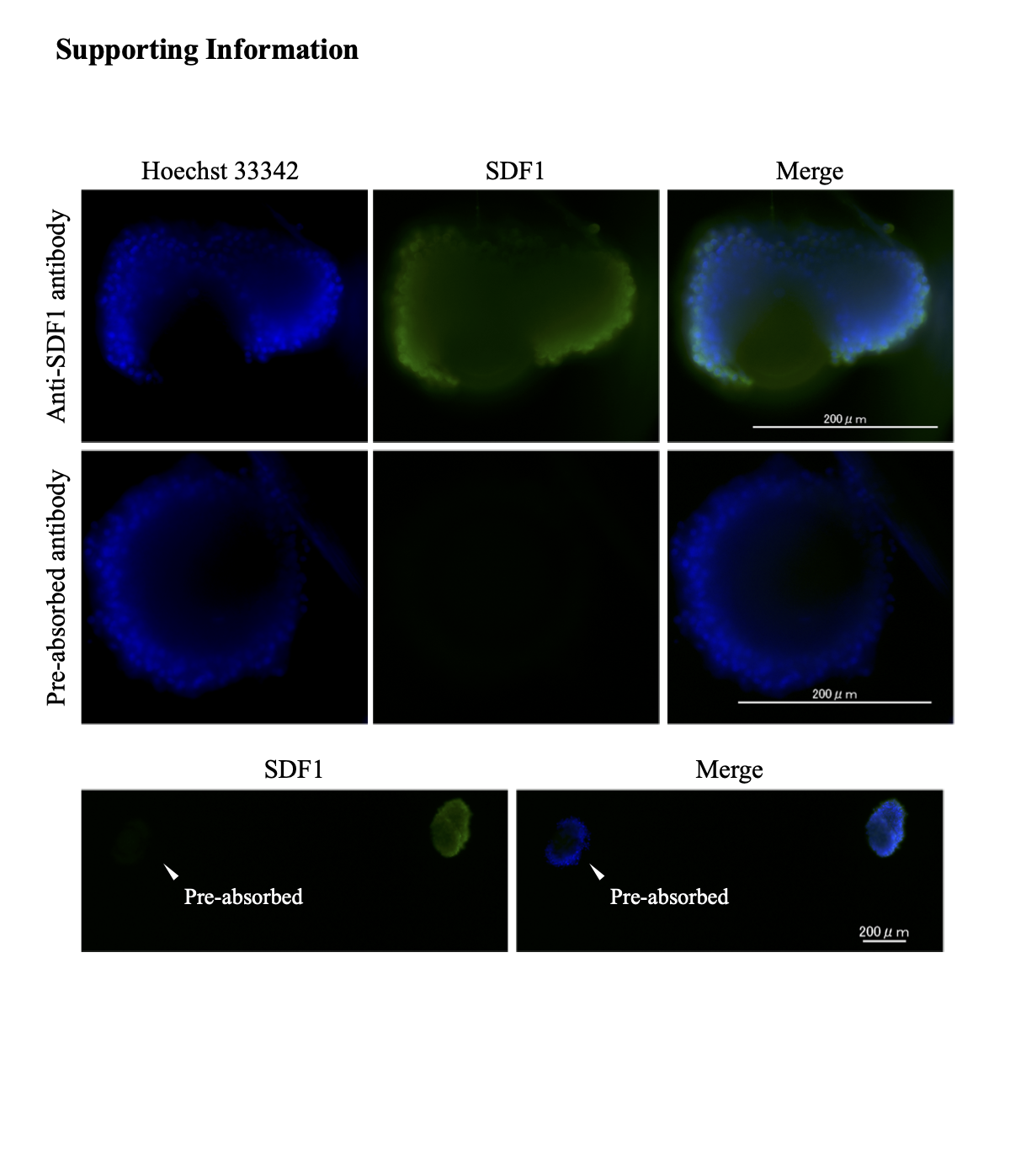

Supplement: S1 Fig — Immunostaining of bovine COCs using an anti-SDF1 antibody pre-absorbed with a 10-fold excess of the epitope-blocking peptide (ab9798; abcam) overnight at 4°C. Blue: nuclei (Hoechst 33342); Green: SDF1. Bars = 200 μm. (TIFF) [file pone.0232536.s001.tiff]

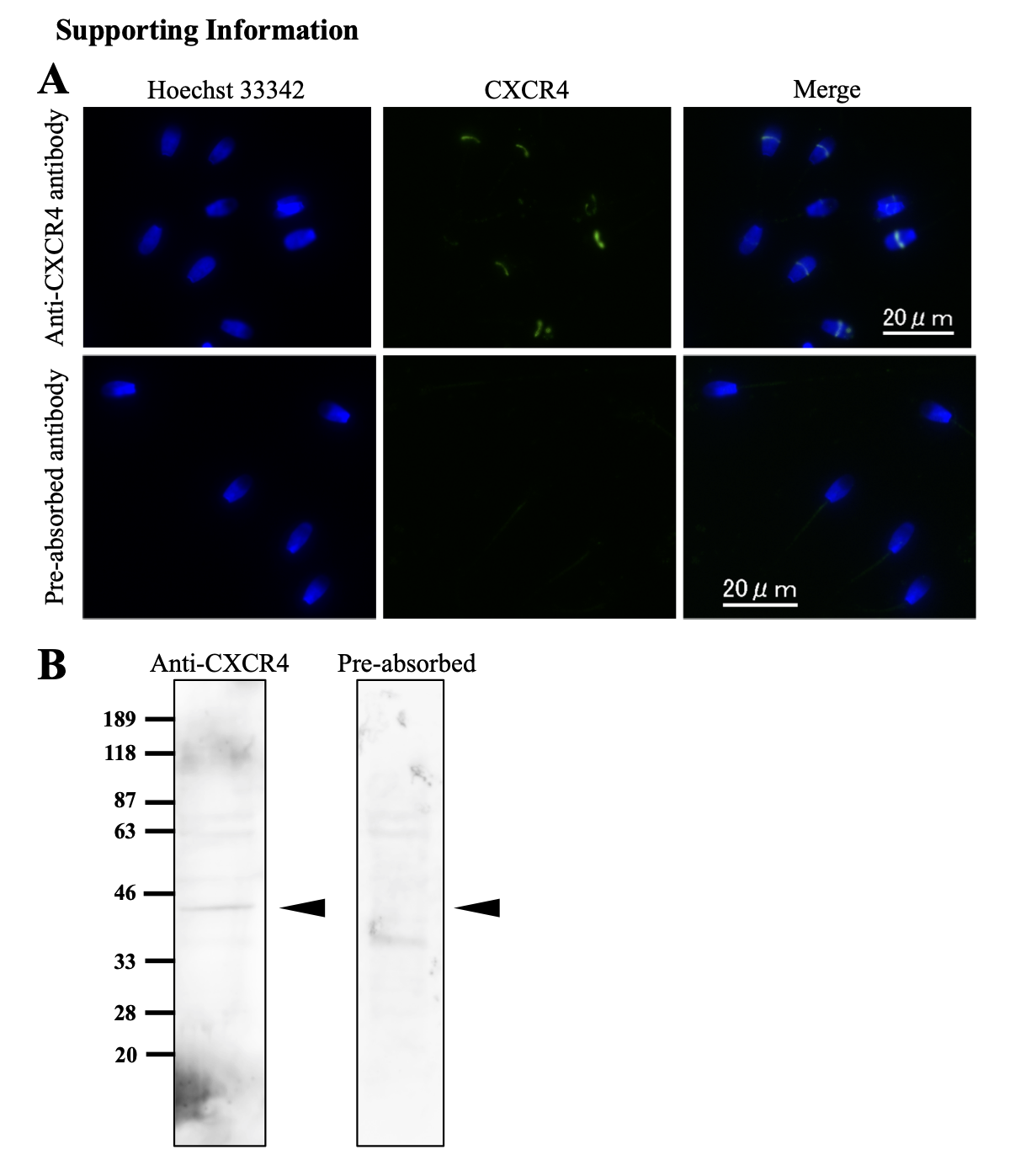

Supplement: S2 Fig — (A) Immunostaining of bovine sperm using an anti-CXCR4 antibody pre-absorbed with a 10-fold excess of the epitope blocking peptide (ab155072; abcam) overnight at 4°C. Blue: nuclei (Hoechst 33342); Green: CXCR4. Bars = 20 μm. (B) Western blotting of sperm proteins with the anti-CXCR4 antibody. Extracted sperm proteins (10 μg) were separated by 10% SDS-PAGE and transferred to a PVDF membrane. After blocking, the primary antibody diluted at 1:500 with a 10-fold excess of the blocking peptide was added as a negative control. The antibody reacted with a 39 kDa protein, which was abolished with the pre-absorbed antibody (arrowhead). (TIFF) [file pone.0232536.s002.tiff]

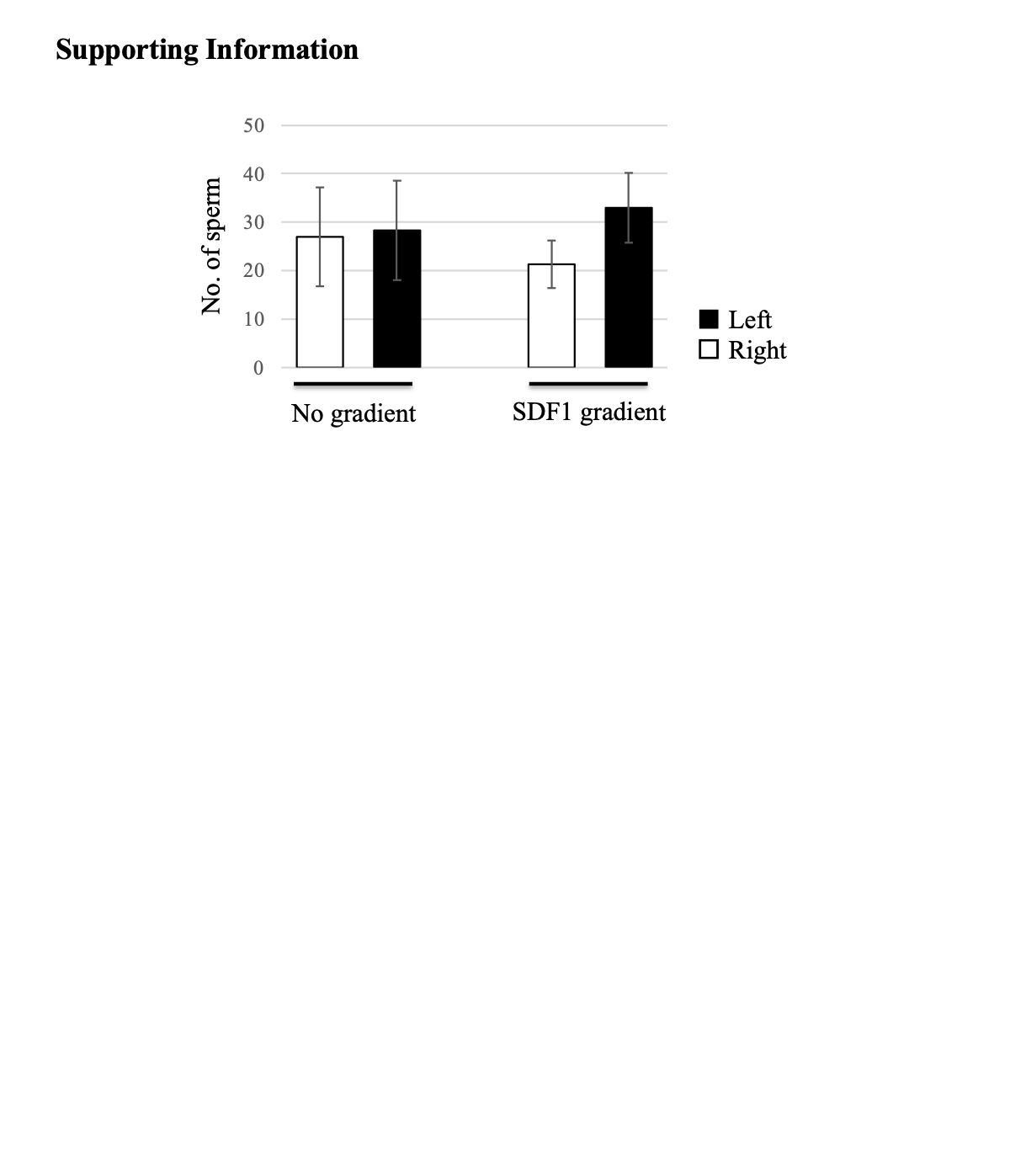

Supplement: S3 Fig — The total number of sperm migrated to the left or right reservoir during 5min of observation. White bars show the number for the sperm migrated to the left reservoir (lower SDF1 concentrations), and black bars show those for the sperm migrated to the right reservoir (higher SDF1 concentrations). Data are shown as the mean ± SE. (TIFF) [file pone.0232536.s003.tiff]

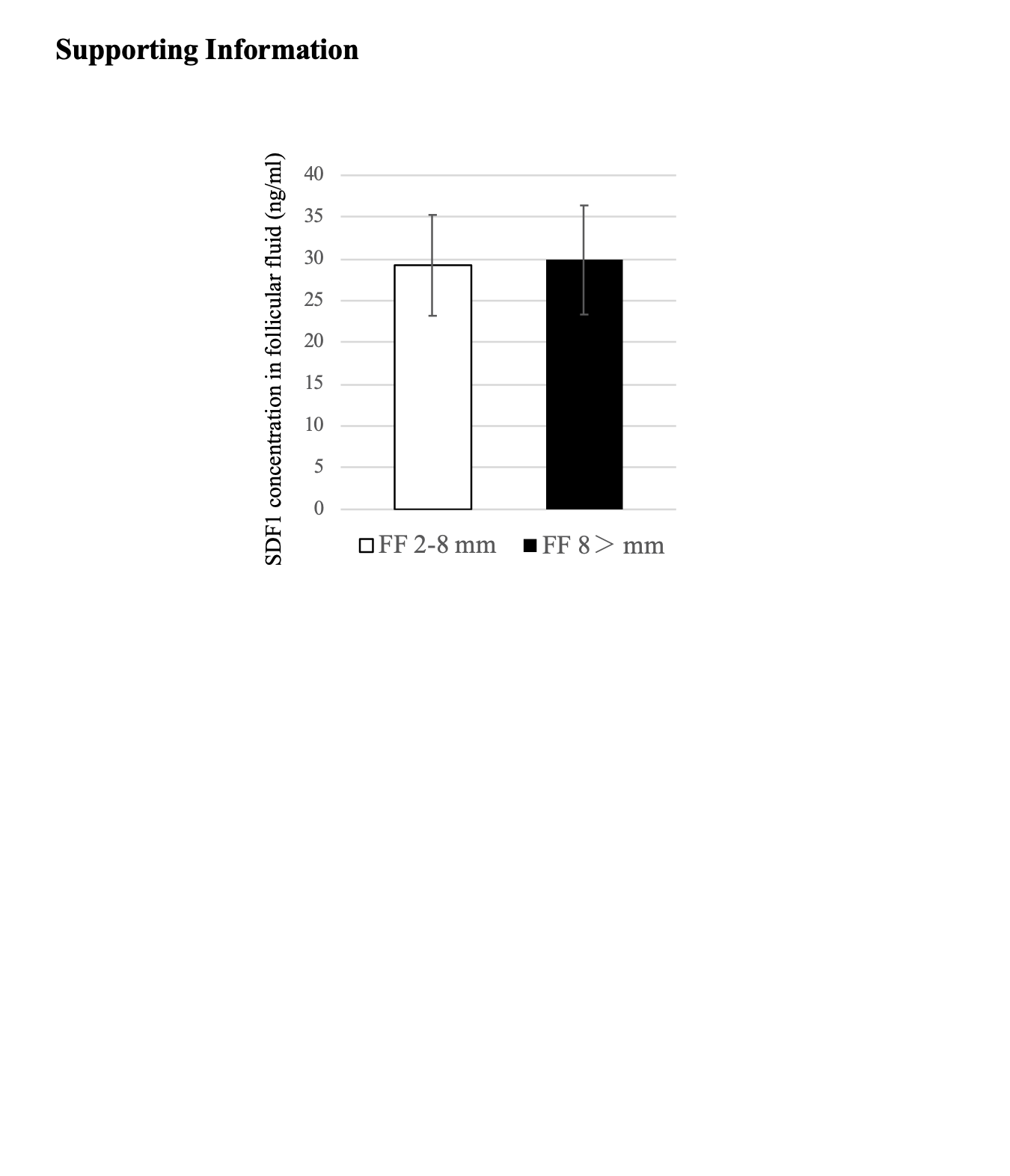

Supplement: S4 Fig — SDF1 quantification in bovine follicular fluid was performed by an ELISA (Bovine Stromal Cell Derived Factor 1 ELISA Kit 96-Strip-Wells Cat. No. MBS741957; MyBiosource, Inc., San Diego, CA, USA), following the manufacturer's instructions. Follicular fluid was collected from follicles of 2–8 mm in diameter or follicles with a diameter of >8 mm of diameter. Data are shown as the mean ± SE. (TIFF) [file pone.0232536.s004.tiff]

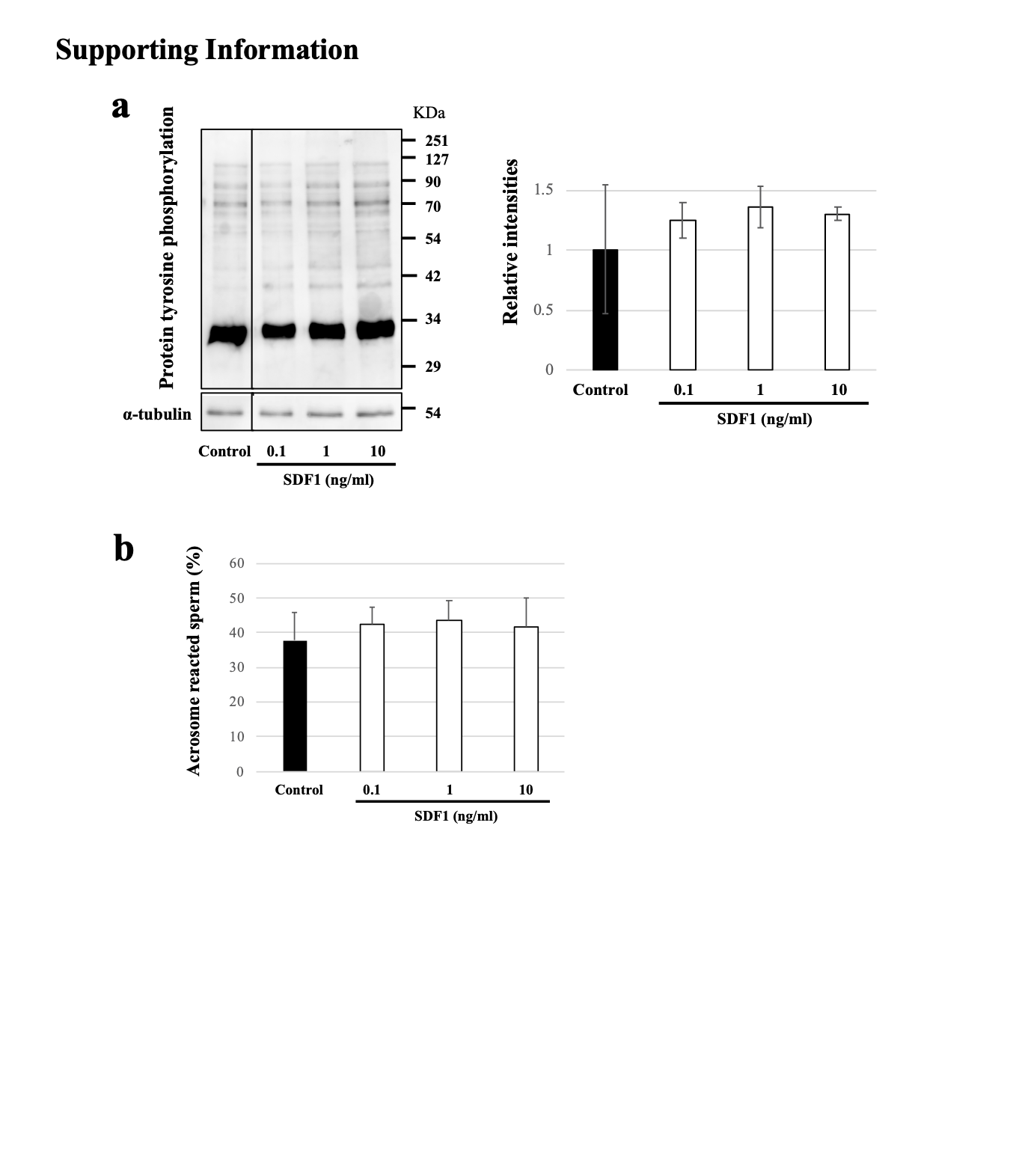

Supplement: S5 Fig — Sperm protein tyrosine phosphorylation, which is the major indicator of sperm capacitation, was evaluated by western blotting. Total tyrosine phosphorylation level was not significantly affected by SDF1 addition, suggesting that SDF1 don’t induce sperm capacitation in bull (S2A Fig). The effect of SDF1 on the acrosome reaction in capacitated sperm was also evaluated. After 4 hours of sperm incubation in heparin-containing BGM-1 medium, to induce capacitation, the acrosome status of sperm was determined by staining with FITC-PNA lectin. The rates of acrosome reacted sperm were not affected by SDF1 addition, suggesting that SDF1 don’t induce acrosome reaction (S2B Fig). Data are shown as the mean ± SE. (TIFF) [file pone.0232536.s005.tiff]

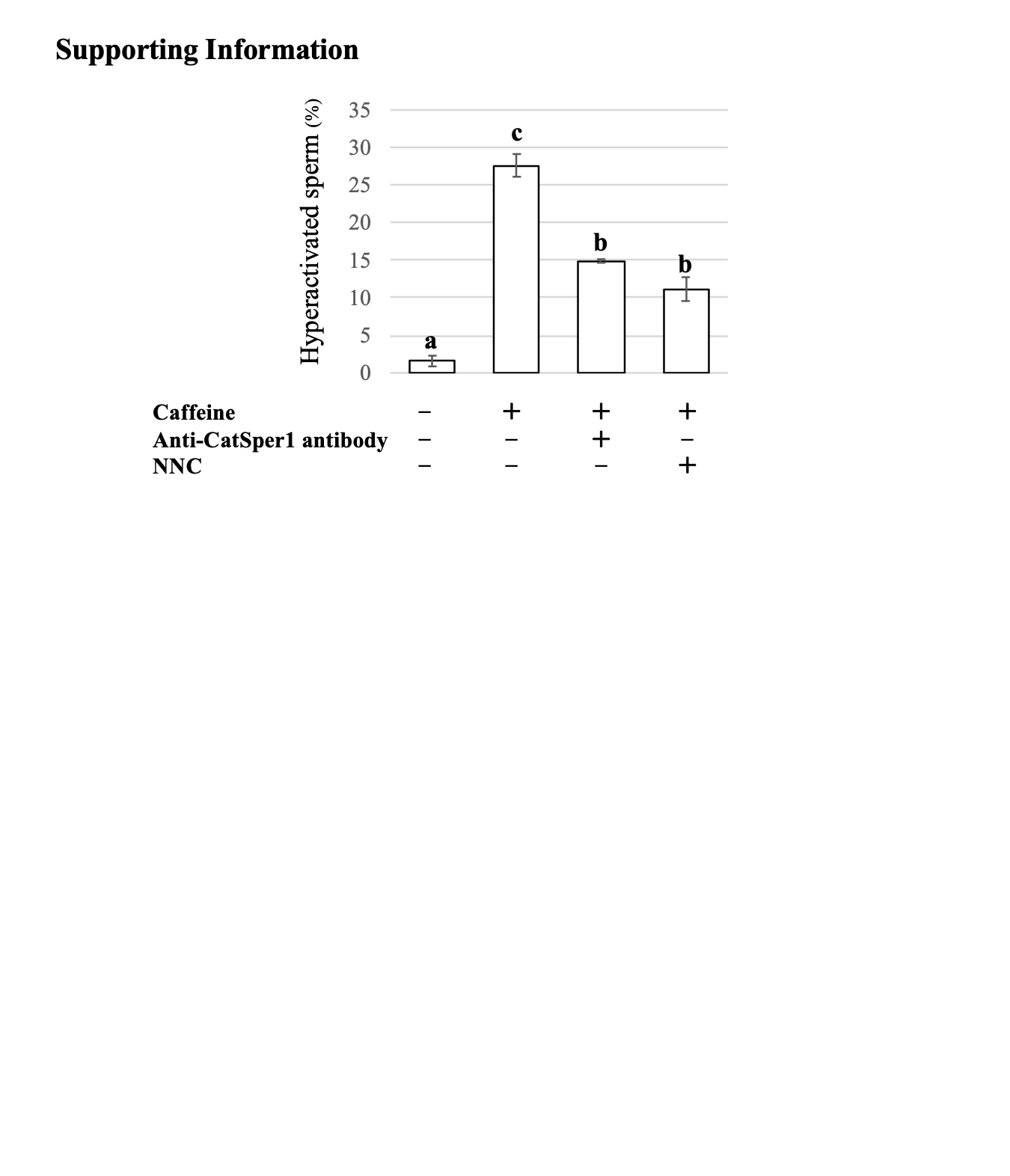

Supplement: S6 Fig — After equal volumes of the sperm suspension in BGM-1 medium were divided, the caffeine, anti-CatSper1 antibody, and/or NNC were added to each suspension at a final concentration of 10 mM, 38 μg/ml, 10 μM, respectively. The samples were placed onto 2-chamber slides with a depth of 12 μm, and observed by using an inverted microscope. At least 100 sperm of each sample were divided into motile and dead sperm, and the percentages of hyperactivated sperm per total motile sperm were calculated. Data are shown as the mean ± SE. Different letters indicate significant difference (p<0.05). (TIFF) [file pone.0232536.s006.tiff]

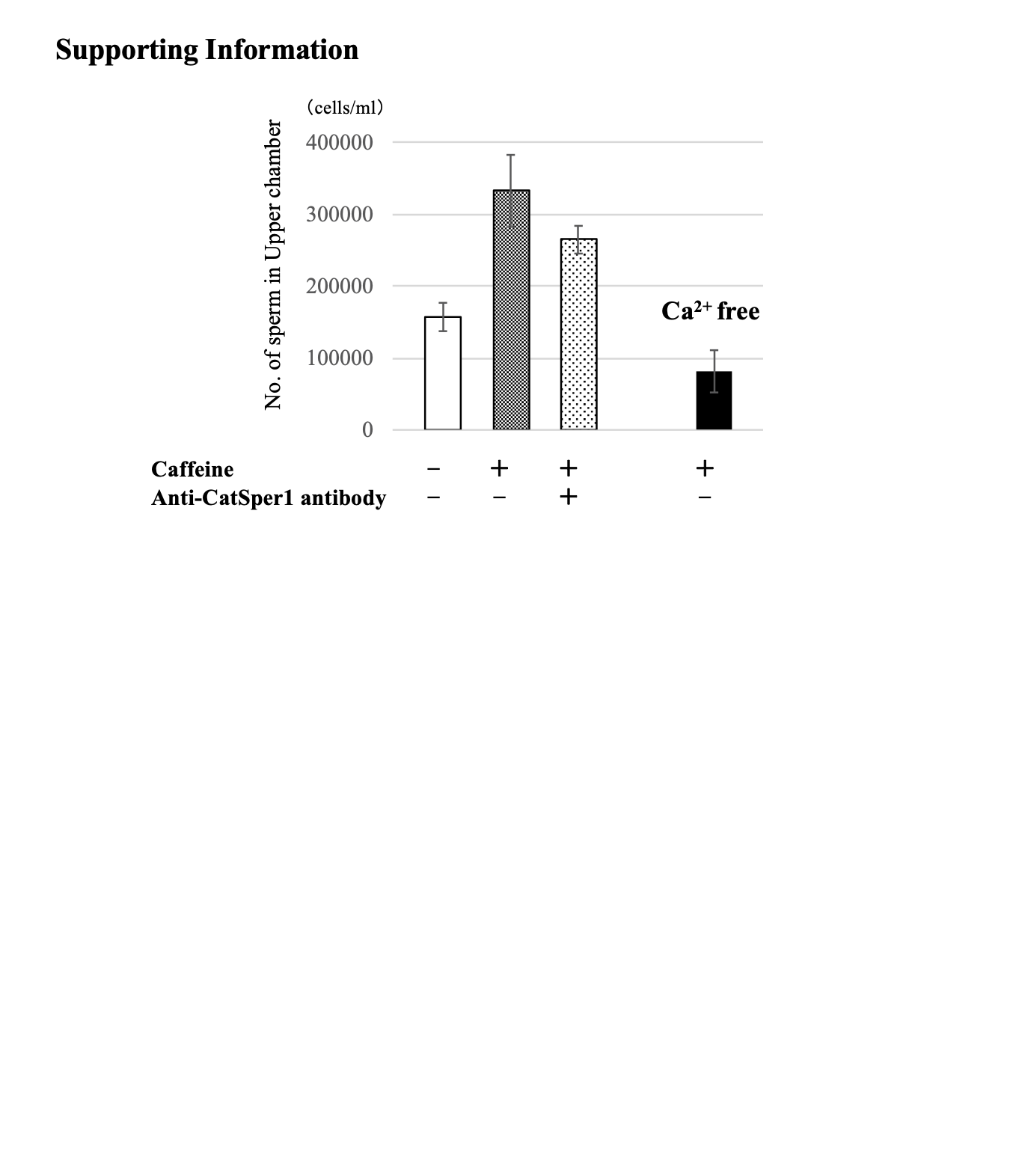

Supplement: S7 Fig — Before the chemotaxis assay, sperm were incubated for 30 min with or without 38 μg/ml of anti-CatSper1 antibody. The lower chamber was filled with each sperm, and the upper chamber was filled with the medium supplemented with 1 ng/ml SDF1. The chamber was incubated for 30 min, and the number of sperm in the upper chamber was calculated. As for Ca2+ free medium, we omitted Ca2+ from the both lower and upper chamber, and conducted the assay in the same way. Data are shown as the mean ± SE. (TIFF) [file pone.0232536.s007.tiff]

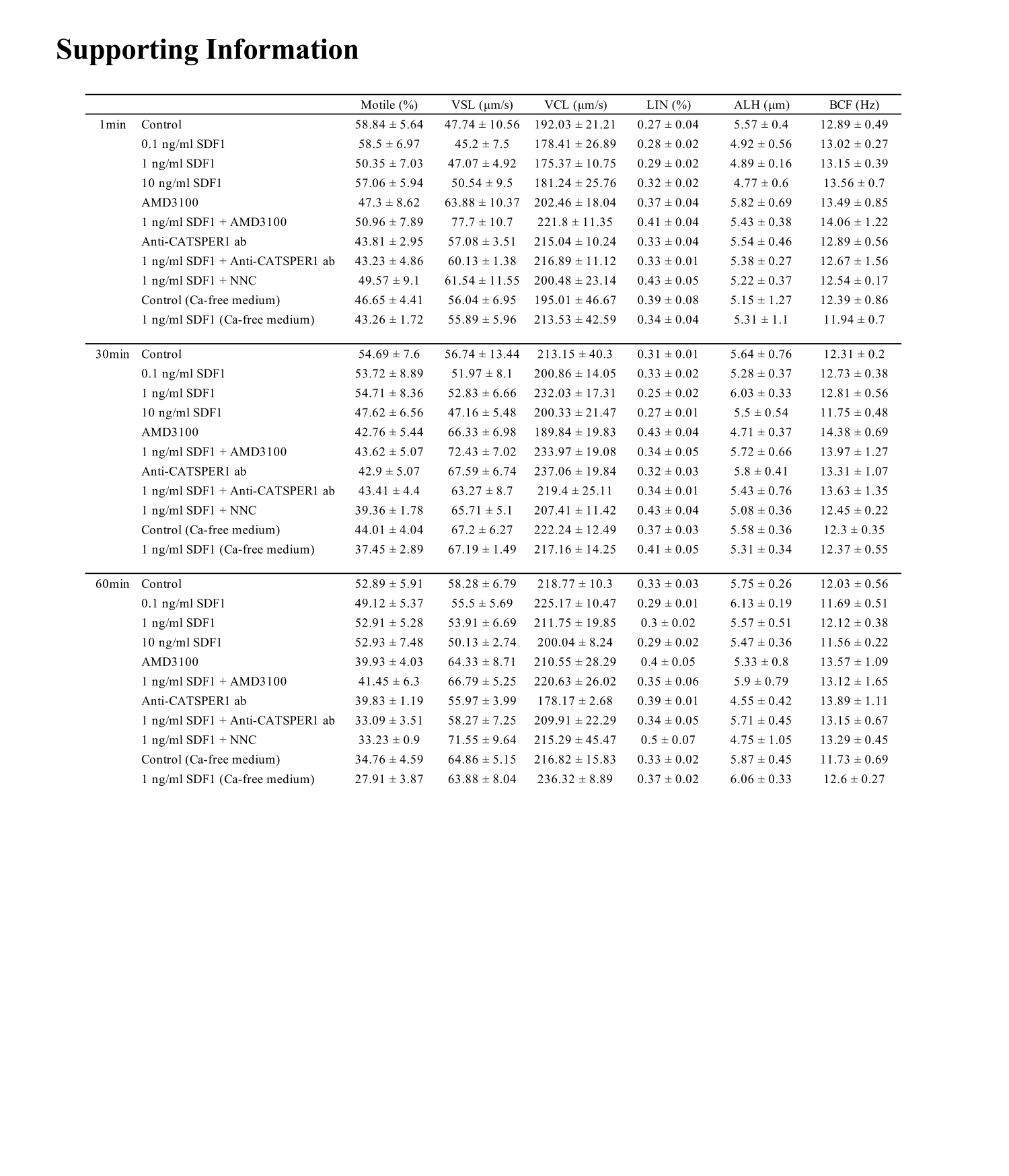

Supplement: S1 Table — VSL, straight-line velocity; VCL, curvilinear velocity (μm/sec); LIN, linearity; ALH, amplitude of lateral head displacement; BCF, beat-cross frequency. Data are shown as the mean ± SE. (TIFF) [file pone.0232536.s008.tiff]

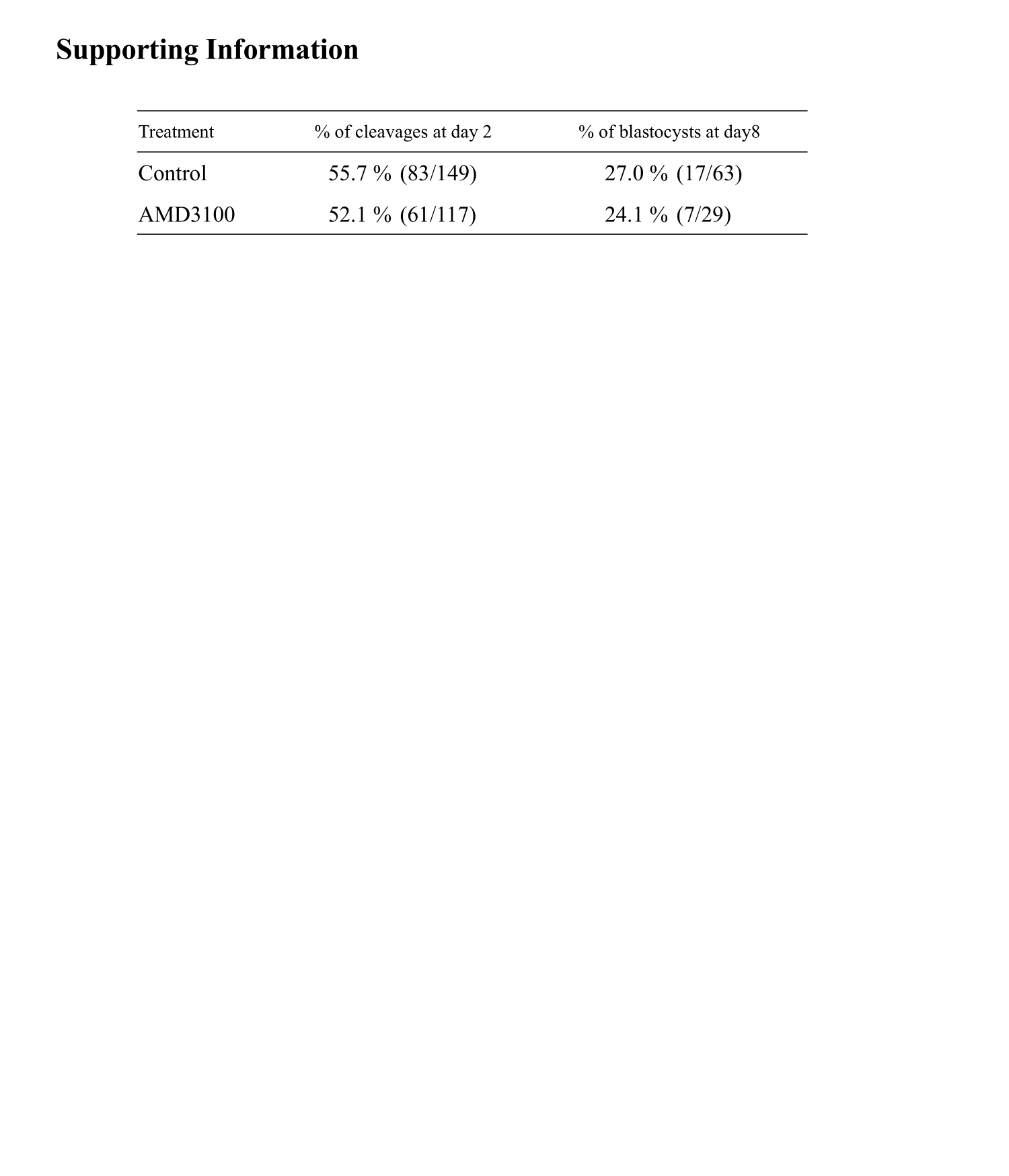

Supplement: S2 Table — Cleavage rate and blastocyst formation rate were assessed at 72 h and 192 h, respectively, after IVF. Cleavage rates and blastocyst formation rates were based on the number of original oocyte number and cleaved oocyte number, respectively. (TIFF) [file pone.0232536.s009.tiff]

**Supporting Information**

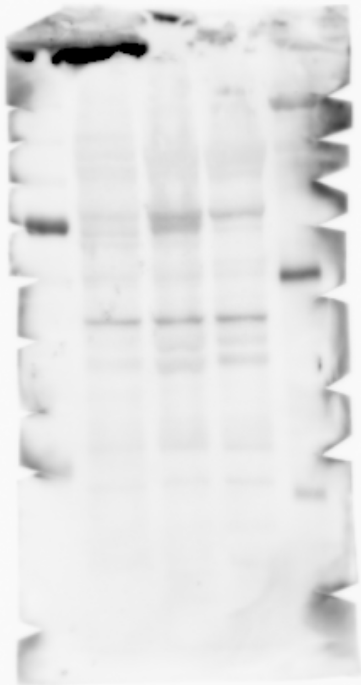

**S1 raw images for Fig. 2C.**

Supplement: S1 Raw images — (PDF) [file pone.0232536.s011.pdf]

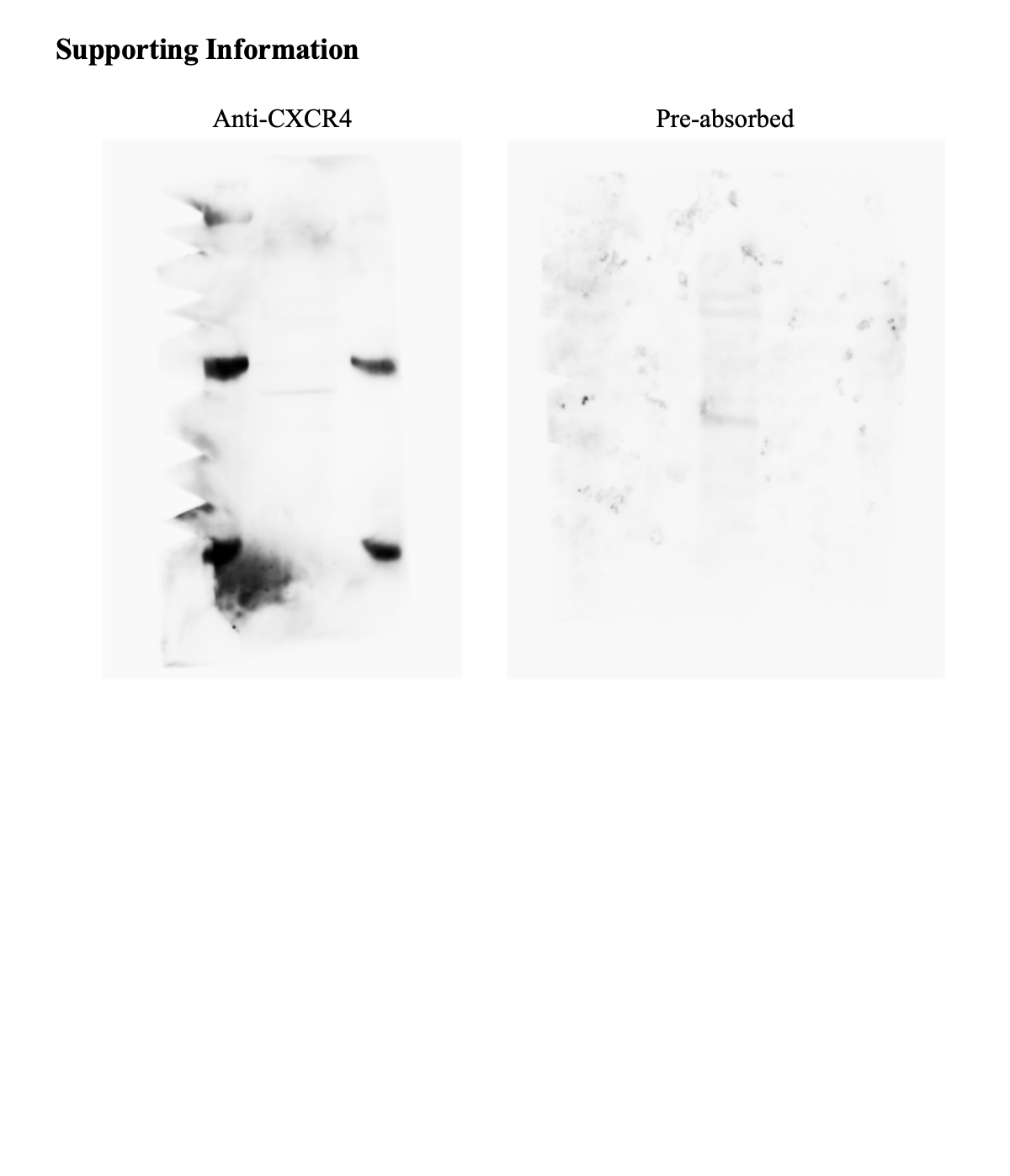

Supplement: S2 Raw images — (TIFF) [file pone.0232536.s012.tiff]

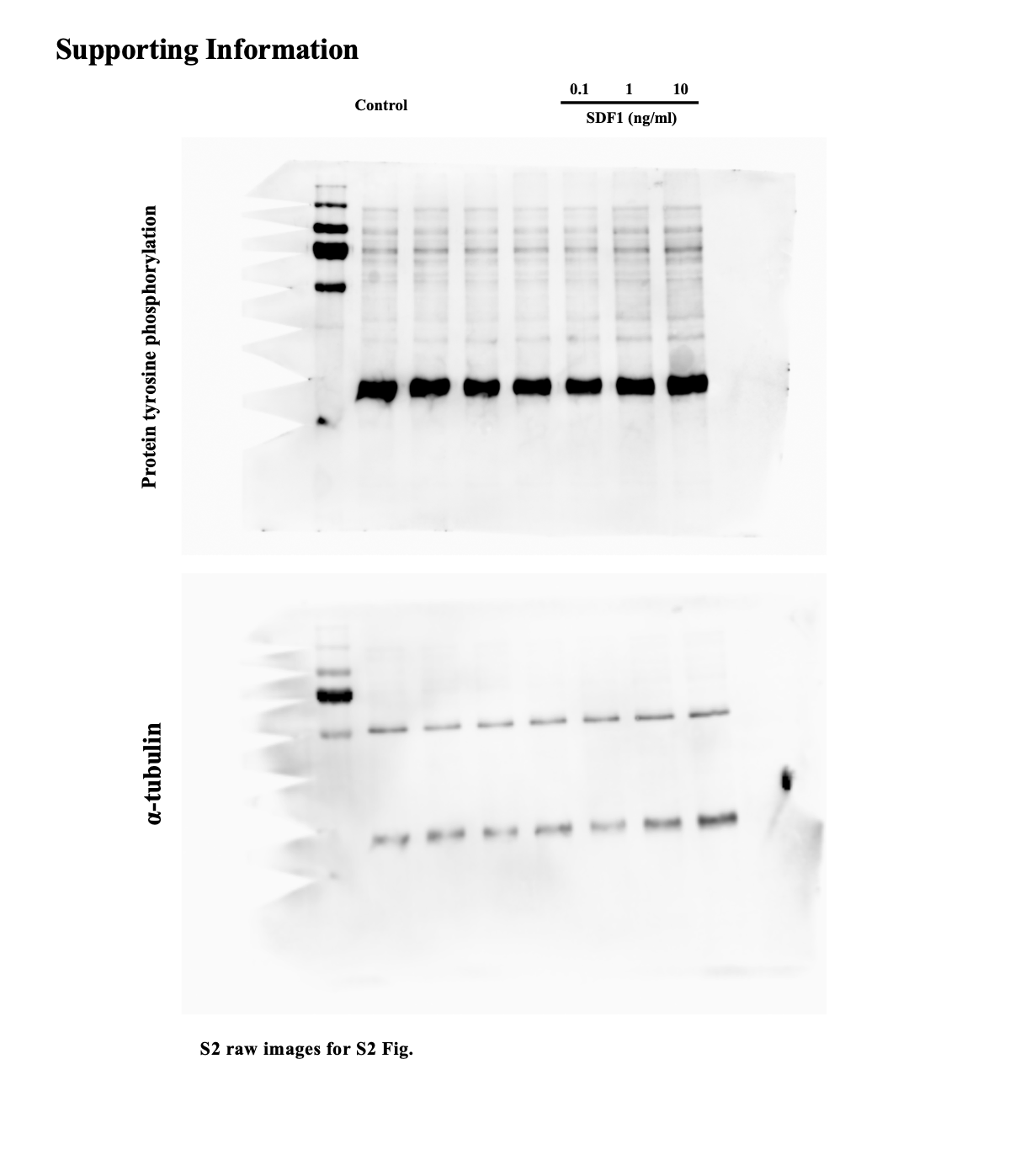

Supplement: S3 Raw images — (TIFF) [file pone.0232536.s013.tiff]
